# Comparison of the antifibrotic effects of the pan-histone deacetylase-inhibitor panobinostat versus the IPF-drug pirfenidone in fibroblasts from patients with idiopathic pulmonary fibrosis

**DOI:** 10.1371/journal.pone.0207915

**Published:** 2018-11-27

**Authors:** Martina Korfei, Daniel Stelmaszek, BreAnne MacKenzie, Sylwia Skwarna, Shashipavan Chillappagari, Anna C. Bach, Clemens Ruppert, Shigeki Saito, Poornima Mahavadi, Walter Klepetko, Ludger Fink, Werner Seeger, Joseph A. Lasky, Soni S. Pullamsetti, Oliver H. Krämer, Andreas Guenther

**Affiliations:** 1 Department of Internal Medicine, Justus-Liebig-University Giessen, Giessen, Germany; 2 Universities of Giessen and Marburg Lung Center (UGMLC), Member of the German Center for Lung Research (DZL), Giessen, Germany; 3 Excellence Cluster Cardio-Pulmonary System (ECCPS), Giessen, Germany; 4 Department of Medicine, Section of Pulmonary Diseases, Critical Care and Environmental Medicine, Tulane University Health Sciences Center, New Orleans, Louisiana, United States of America; 5 Department of Thoracic Surgery, Vienna General Hospital, Vienna, Austria; 6 European IPF Network and European IPF Registry, Giessen, Germany; 7 Institute of Pathology and Cytology, Wetzlar, Germany; 8 Max-Planck-Institute for Heart and Lung Research, Department of Lung Development and Remodeling, Bad Nauheim, Germany; 9 Department of Toxicology, University Medical Center, Mainz, Germany; 10 Agaplesion Lung Clinic Waldhof Elgershausen, Greifenstein, Germany; Universitatsklinikum Freiburg, GERMANY

## Abstract

**Background:**

Idiopathic pulmonary fibrosis (IPF) is a devastating lung disease with a poor prognosis. Pirfenidone is the first antifibrotic agent to be approved for IPF-treatment as it is able to slow down disease progression. However, there is no curative treatment other than lung transplantation. Because epigenetic alterations are associated with IPF, histone deacetylase (HDAC)-inhibitors have recently been proven to attenuate fibrotic remodeling *in vitro* and *in vivo*. This study compared the effects of pirfenidone with the pan-HDAC-inhibitor panobinostat/LBH589, a FDA-approved drug for the treatment of multiple myeloma, head-to-head on survival, fibrotic activity and proliferation of primary IPF-fibroblasts *in vitro*.

**Methods:**

Primary fibroblasts from six IPF-patients were incubated for 24h with vehicle (0.25% DMSO), panobinostat (LBH589, 85 nM) or pirfenidone (2.7 mM), followed by assessment of proliferation and expression analyses for profibrotic and anti-apoptosis genes, as well as for ER stress and apoptosis-markers. In addition, the expression status of all HDAC enzymes was examined.

**Results:**

Treatment of IPF-fibroblasts with panobinostat or pirfenidone resulted in a downregulated expression of various extracellular matrix (ECM)-associated genes, as compared to vehicle-treated cells. In agreement, both drugs decreased protein level of phosphorylated (p)-STAT3, a transcription factor mediating profibrotic responses, in treated IPF-fibroblasts. Further, an increase in histone acetylation was observed in response to both treatments, but was much more pronounced and excessive in panobinostat-treated IPF-fibroblasts. Panobinostat, but not pirfenidone, led to a significant suppression of proliferation in IPF-fibroblasts, as indicated by WST1- and BrdU assay and markedly diminished levels of cyclin-D1 and p-histone H3. Furthermore, panobinostat-treatment enhanced α-tubulin-acetylation, decreased the expression of survival-related genes Bcl-XL and *BIRC5*/survivin, and was associated with induction of ER stress and apoptosis in IPF-fibroblasts. In contrast, pirfenidone-treatment maintained Bcl-XL expression, and was neither associated with ER stress-induction nor any apoptotic signaling. Pirfenidone also led to increased expression of HDAC6 and sirtuin-2, and enhanced α-tubulin-deacetylation. But in line with its ability to increase histone acetylation, pirfenidone reduced the expression of HDAC enzymes HDAC1, -2 and -9.

**Conclusions:**

We conclude that, beside other antifibrotic mechanisms, pirfenidone reduces profibrotic signaling also through STAT3 inactivation and weak epigenetic alterations in IPF-fibroblasts, and permits survival of (altered) fibroblasts. The pan-HDAC-inhibitor panobinostat reduces profibrotic phenotypes while inducing cell cycle arrest and apoptosis in IPF-fibroblasts, thus indicating more efficiency than pirfenidone in inactivating IPF-fibroblasts. We therefore believe that HDAC-inhibitors such as panobinostat can present a novel therapeutic strategy for IPF.

## Introduction

Idiopathic pulmonary fibrosis (IPF) is a devastating interstitial lung disease of unknown origin with a poor prognosis. It predominantly affects individuals aged 60 to 75 years old, with a median survival of 3–5 years after diagnosis, which is akin to many aggressive cancers [1,2]. Although pirfenidone (Esbriet^®^) and nintedanib (Ofev^®^) have recently been approved as IPF therapies, with indication of manageable side effect profiles, both drugs only slow down the progression of the disease [3–5]. Therefore, there is no curative treatment other than lung transplantation.

The current pathogenic model of IPF suggests that lung fibrosis develops as a result of repetitive injurious insults in combination with genetic and aging-related risk factors to type-II alveolar epithelial cells (AECII). Consecutively, an aberrant wound healing response is triggered through activation of fibroblasts and myofibroblasts and replacement of injured alveolar epithelium with fibrotic scar tissue, resulting in irreversible lung damage and an inevitable decline in respiratory function [1,2,6–8]. It is suggested that soluble molecules released from injured AECII such as cytokines, chemokines and growth factors or other mediators activate fibroblast proliferation, migration and transformation of fibroblasts into myofibroblasts [9,10]. Myofibroblasts are contractile protein-expressing cells and characterized by *de novo* expression of alpha-smooth muscle actin (α-SMA) [11]. In IPF, myofibroblasts progressively expand in the lung interstitium and accumulate in fibroblastic foci (FF) which are specific aggregates responsible for the exaggerated deposition of collagen and other ectracellular matrix (ECM) compounds in the lung parenchyma [1,2,6].

Multiple signaling pathways and their downstream targets have been reported to be implicated in the persistent activation of fibroblastic cell populations in IPF. Overwhelming evidence supports a key role for transforming growth factor beta-1 (TGF-β1) in driving these processes, via activation of the canonical TGF-β-SMAD signaling pathway through TGF-β receptors (TGF-βRs) [12,13]. In addition, studies in mouse models of pulmonary fibrosis indicate that inhibition of mitogen activated protein kinases (MAPK) and/or phosphatidylinositide 3-kinase (PI3K) pathways attenuates the development of lung fibrosis [14,15]. It was also found that 17-N-allylamino-17-demethoxygeldanamycin (17-AAG), a small-molecule inhibitor of Hsp90, inhibited TGF-β1-induced myofibroblast transformation and ECM production in primary lung fibroblasts *in vitro* and abolished significantly myofibroblast accumulation, ECM deposition and fibrotic tissue generation in the bleomycin- as well as TGFα mouse model of pulmonary fibrosis *in vivo* [13,16]. In addition, it has been shown that locally produced and circulation-derived FII (thrombin) as well as FXa can induce profibrotic effects via proteolytic activation of protease-activated receptor-1 (PAR1) and subsequent differentiation of fibroblasts into myofibroblast [17,18]. Several studies indicate also a significant role of phosphorylated, activated STAT3 (signal transducer and activator of transcription 3), which can be induced by TGF-β1 as well as IL-6 family of cytokines, in fibrogenesis in IPF [19,20]. It could be demonstrated that C-188-9, a small molecule STAT3 inhibitor, which targets the phospho-Tyr705 peptide binding pocket, decreased experimental pulmonary fibrosis in mice, as shown by dimished α-SMA expression and reduced collagen deposition [20].

At present, the FDA approved drugs nintedanib (Ofev^®^) and pirfenidone (Esbriet^®^) are widely used for IPF therapy [5,21]. Nintedanib is a receptor tyrosine kinase inhibitor of platelet-derived growth factor receptor (PDGFR)-, vascular endothelial growth factor receptor (VEGFR)- and fibroblast growth factor receptor (FGFR) signaling, which have been shown to critically regulate myofibroblast transformation and collagen production under fibrotic conditions, through subsequent signaling via the ERK, MAPK and the PI3K/Akt pathways [22–24].

The direct targets of pirfenidone are unknown, but it elicits significant anti-fibrotic, anti-inflammatory and anti-oxidant effects in experimental models of lung fibrosis [25–27]. The antifibrotic property of pirfenidone is demonstrated to depend on its ability to inhibit the direct production of profibrotic cytokines and growth factors, such as TGF-β1, basic-FGF, PDGF, Interleukin 1β (IL-1β), and TNFα in these models [25–29]. In addition, pirfenidone has been reported to interfere with insufficient mitophagy and consequent myofibroblast differentiation in fibrotic fibroblasts *in vitro* and *in vivo*, through inducing autophagy/mitophagy via enhanced expression of E3 ubiquitin ligase PARK2 and suppressing mitochondrial ROS production and PDGFR-PI3K/Akt signaling [30]. Although more than one pathway or cellular process of fibroblast activation can be blocked by nintedanib or pirfenidone, IPF cannot be cured with these drugs.

We and others have reported that IPF-fibroblasts exhibit a cancer-like phenotype due to aberrant overexpression of class-I and class-II histone deacetylase (HDAC) enzymes, which appeared to be responsible for their abnormal activation and persistence in IPF, presumably as the result of alterations in the acetylation status of the chromatin and various non-histone proteins [31–33]. In accordance, we could demonstrate that the pan-HDAC inhibitor panobinostat (LBH589) reduced proliferation, collagen-I biosynthesis, and anti-apoptotic genes in IPF-fibroblasts *in vitro*, with concomitant induction of ER stress-mediated apoptosis [31]. In addition, Sanders and coworkers demonstrated amelioration of pulmonary fibrosis in response to global HDAC inhibition by SAHA (vorinostat) in bleomycin-treated mice *in vivo* [33].

Panobinostat (Farydak^®^) was developed by Novartis for the treatment of various cancers, and in February 2015 it received accelerated approval by the US Food and Drug Administration (FDA) for treatment of adult patients with multiple myeloma. In August 2015 it was approved by the European Medicines Agency for the same use [34–36]. Currently, this neoplastic drug is along with other HDAC inhibitors also being studied in patients with HIV for potential to affect latent HIV viral reservoirs, as it has demonstrated effective disruption of HIV latency in these patients [37].

We were interested to compare the anti-fibrotic efficacy of panobinostat/LBH589 with the IPF drug pirfenidone, in order to evaluate the potential use of panobinostat as additional, future IPF therapy. Because attenuation of lung fibrosis by pirfenidone has been associated with reduced expression of profibrotic cytokines, we were also interested in examining its effect on histone-acetylation and HDAC activity in cultured, primary IPF-fibroblasts.

## Material and methods

### Human lung tissue

Peripheral lung tissue samples were obtained from explanted lungs of 6 patients with sporadic IPF (mean age ± SD: 50.10 ± 16.64 years; 2 females, 4 males), and used for fibroblast isolations and consecutive biochemical research, as described below. Additionally, formalin-fixed, paraffin-embedded lung tissue samples from 5 patients with sporadic IPF (mean age ± SD: 57.00 ± 10.89 years; 1 female, 4 males) and 5 non-diseased control subjects (organ donors; mean age ± SD: 50.40 ± 17.01 years; 3 females, 2 males) were immunohistochemically researched. All described IPF-patients did not previously receive pirfenidone-treatment. All lung tissue samples were collected in frame of the European IPF registry (eurIPFreg) and provided by the UGMLC Giessen Biobank (member of the DZL Platform Biobanking). The study protocol was approved by the Ethics Committee of the Justus-Liebig-University Giessen (No. 111/08 and 58/15). All IPF diagnoses were made according to the American Thoracic Society (ATS)/European Respiratory Society (ERS) consensus criteria [1].

### Isolation of primary human lung fibroblasts

Primary human lung fibroblasts were isolated from explanted IPF lungs (n = 6) using an outgrowth-technique as published [31]. Fibroblasts were grown and maintained in MCDB 131 medium (PAN Biotech) containing 10% (v/v) FBS (Sigma), 100 U/mL penicillin, 100 μg/mL streptomycin, 2 mM L-glutamine (all from Invitrogen), 2 ng/mL basic-FGF (Invitrogen), 0.5 ng/mL EGF (Sigma) and 5 μg/mL insulin (Invitrogen), at 37°C in 5% CO_2_ atmosphere. Experiments were carried out with IPF-fibroblasts between passages 3 and 4.

### Cell culture experiments

Primary fibroblasts of patients with IPF (n = 6) were seeded in normal culture medium as described above, on 10 cm tissue culture dishes and cultured for 3 days at 37°C in 5% CO_2_ atmosphere. At 95–99% confluency, the medium was replaced with culture medium containing 2% (v/v) FBS. IPF-fibroblasts were then incubated for 24h with the HDAC inhibitor panobinostat (LBH589, 85 nM, #S1030, Selleckchem) or the IPF drug pirfenidone (2.7 mM, #P2116, Sigma), and as control experiment with the respective solvent [0.25% (v/v) DMSO, vehicle-control]. The pan-HDACi LBH589 has an efficient inhibitory activity at nanomolar concentrations (10–200 nM) and appears to be the most potent clinically available HDAC inhibitor [38,39]. The dosages of LBH589 or pirfenidone were chosen according to published studies [30,31,38,40–42]. After incubation, fibroblastic cells from each plate were harvested by trypsination and divided in two equal volume parts, and centrifuged using two Falcon-tubes (5 min, 1000 rpm, RT) resulting in two pellets. One pellet was subjected to protein isolation, the other to RNA isolation.

### WST-1 proliferation assay

WST-1 (4- [3- (4- iodophenyl)- 2- (4-nitrophenyl)- 2H- 5-tetrazolio]- 1,3-benzene disulphonate) (Roche) is a colorimetric assay that quantifies mitochondrial dehydrogenase activity and thus reflects cell viability. In brief, IPF-fibroblasts (n = 4) were seeded in 96-well plates (2×10^4^ cells/200 μL/well) and were allowed to grow overnight in normal culture medium at 37°C in 5% CO_2_ atmosphere. Next day, the medium was replaced, and fibroblasts were incubated for 24h with vehicle, LBH589 or pirfenidone, as described above. Subsequently, cells were allowed to react with 10 μL/well tetrazolium salt WST-1 and reincubated for 4 h. Thereafter, the number of viable cells was quantified based on the absorbance of formazan dye formed at 450 nm in an ELISA microtiter plate reader (SpectraFluor Plus, Tecan). The assay was performed in triplicates.

### Colorimetric bromodeoxyuridine (BrdU) assay

The colorimetric bromodeoxyuridine (BrdU) assay (Roche/Sigma-Aldrich) is based on incorporation of BrdU into newly synthesized DNA in proliferating cells, and quantitative binding of a monoclonal HRP-coupled anti-BrdU-antibody. In brief, IPF-fibroblasts (n = 6) were seeded in 96-well plates (1×10^4^ cells/200 μL/well) and were allowed to grow overnight in normal culture medium. Next day, the medium was replaced, and fibroblasts were incubated for 24h with vehicle, LBH589 or pirfenidone, as described above. Subsequently, fibroblastic cells were labeled with 10 μM BrdU and re-incubated for 12h, followed by fixation (30 min/RT) and incubation with HRP-coupled anti-BrdU-antibody for 90 min at RT. Thereafter, 100 μL substrate solution (tetramethyl-benzidine) per well was added, and the plate was incubated for 5–25 min at RT until color development (blue) was sufficient. The substrate reaction was then stopped by adding 25 μL 1M H_2_SO_4_ per well and thorough mixing (yellow color). Immediately, absorbance was measured at 450 nm in an ELISA microtiter plate reader. The assay was performed in triplicates.

### Reverse transcription-polymerase chain reaction (RT-PCR)

RT-PCR for *ACTA2*, *COL1A1*, *COL3A1*, *FN*, *CNN1*, *DES*, *P4HTM*, *CCND1*, *BIRC5*, *CIP1*, *P53*, *CDKN2A*, *PUMA*, *DR5*, *ATF6*, *CHOP*, *HDACs1-11*, *SIRT1* and *SIRT2* was done in vehicle-, panobinostat (LBH589)- and pirfenidone-treated IPF-fibroblasts, as previously described [31]. *GAPDH* was used as reference gene. Full details for RT-PCR including agarose gel electrophoresis and quantification of PCR products using Image Lab-Software (version 5.2.1, Bio-Rad), are available in the Supporting Information [S1 Text: Supplementary Methods, S1 Table: list of gene-specific primers (homo sapiens), including the size of the amplified PCR product for each gene/*c*DNA, and the number of cycles for amplification of each *c*DNA].

### Western blot

Protein expression of histone H3-acetyl K27, acetylated α-tubulin, phospho-histone H3 (Ser10), cyclin D1, p-STAT3 (Tyr705), α-SMA, tropomyosin, collagen-I, HDAC1, HDAC2, HDAC6, HDAC9, sirtuin-2, survivin, Bcl-XL, CHOP, p21, p53, p16, AIF (mitochondrial), DR5, cleaved PARP1-p25 and cleaved caspase-3 in vehicle-, panobinostat (LBH589)- and pirfenidone-treated IPF-fibroblasts was analyzed by quantitative immunoblotting of cell lysates, as described before [31]. Dependent on research target, the expression of histone H3, STAT3, GAPDH or β-actin served as loading control after stripping of blots. Detection of primary antibodies bound to protein targets on PVDF membranes was done with respective horseradish peroxidase (HRP)-conjugated secondary antibodies (DakoCytomation, Hamburg, Germany; rabbit anti-mouse-IgG, rabbit anti-goat-IgG, or swine anti-rabbit IgG). Blot membranes were developed with the Immobilon Western Chemiluminescent HRP substrate (Millipore), and emitted signals were detected with a chemiluminescence imager (Intas ChemoStar, Germany). For quantification, band intensities were quantified by densitometry using ImageJ software (Version 1.46r, NIH). The band densities were normalized to loading controls. Full details for western blot method, including the sources and dilutions of all employed primary antibodies, are available in the Supporting Information. (S1 Text: Supplementary Methods).

### Immunohistochemistry (IHC)

ZytoChem-Plus AP Kit (Fast Red) (Zytomed Systems, Berlin, Germany) was used for immunohistochemical localization of research target-proteins in formalin-fixed, paraffin-embedded lung tissue sections from patients with sporadic IPF (n = 5) and organ donors (n = 5), according to the manufacturer´s instructions and previous published work [31]. In the following, the primary antibodies used for IHC are listed, including the sources and dilutions: rabbit polyclonal for human alpha-smooth muscle actin [α-SMA] (1:200, Abcam, ab5694), rabbit monoclonal for human cytokeratin-5 [KRT5] (1:200, Abcam, ab75869), rabbit polyclonal for human survivin (1:200, Abcam, ab24479), mouse monoclonal for human phospho-STAT3 [Y705] (1:25, Cell Signaling Technology, #4113S) and rabbit polyclonal for human HDAC4 (1:50, Santa Cruz, sc-11418). As control experiments, the first antibody was omitted on some sections during staining procedures. Immunostained lung sections were scanned with a scanning device (Nano-Zoomer, Hamamatsu), and examined histopathologically using the ´NDP.view2 software´ at 50×, 100×, 200× and 400× original magnification.

### Apoptosis assay using TUNEL method

The terminal deoxynucleotidyl transferase-mediated dUTP nick end labeling (TUNEL)-reaction is a method for detection and quantification of apoptosis at the single-cell level, based on detection of DNA fragmentation by labeling the 3′- hydroxyl termini in the double-strand DNA breaks generated during apoptosis. In brief, IPF-fibroblasts (n = 4) were seeded in 8-well chamber-slides (2×10^4^ cells/400 μL/well) and were allowed to grow overnight in normal culture medium at 37°C in 5% CO_2_ atmosphere. Next day, the medium was replaced, and fibroblasts were incubated for 24h with vehicle, LBH589 or pirfenidone, as described above. Cells were then fixed with freshly prepared 4% (w/v) paraformaldehyde in 1×PBS for 30 min at RT, followed by permeabilization of cells in a freshly prepared solution containing 0.2% (w/v) Triton X-100 and 0.1% (w/v) sodium citrate, on ice for 15 min. Thereafter, IPF-fibroblasts were incubated for 1h with TUNEL reaction mixture (100 μL/well) containing fluorescein (FITC)-dUTP and the enzyme terminal deoxynucleotidyl transferase (TdT), at 37°C in a humidified atmosphere in the dark. As negative control experiments, cells were incubated with label solution without the TdT enzyme. After extensive washing with 1×PBS, cells were counterstained with DAPI and mounted in Vectashield (Vector Laboratories, Burlingame). The FITC-labeled DNA fragments in the apoptotic cells were visualised using Axio Observer.Z1 fluorescence microscope (Carl Zeiss MicroImaging, Germany).

### Immunofluorescence

Immunofluorescence using mouse monoclonal α-SMA-Cy3™ antibody (#C6198, Sigma-Aldrich) was performed on vehicle-, panobinostat (LBH589)-, and pirfenidone-treated IPF-fibroblasts (n = 3). Pharmacological treatments, fixation and permeabilization of cells were done in 8-well chamber slides as described above. After blocking of cells with 2% (w/v) BSA in 1×PBS, cells were incubated for 90 min with α-SMA-Cy3™ antibody, diluted 1:200 in blocking buffer.

For analyses of F-actin structures, differently treated IPF-fibroblasts (n = 3) were incubated for 10 min with AlexaFluor 555-Phalloidin (Invitrogen, Carlsbad, CA), diluted 1:500 in 1×PBS. Thereafter, cells were counterstained with DAPI and mounted in Vectashield.

### Statistical analysis

All data are presented as median ± range. Data were analyzed by GraphPad Prism 5.02 software. For the statistical comparison of differences between the vehicle- and panobinostat/LBH589 group, the vehicle- and pirfenidone group, and between the pirfenidone- and the panobinostat/LBH589 group, the non-parametric Mann Whitney test was applied. Significance level is indicated by *p<0.05, **p<0.01, ***p < 0.001.

## Results

### Altered acetylation status of histone H3 and α-tubulin in IPF-fibroblasts in response to panobinostat- and pirfenidone-exposure

**Fig 1:**
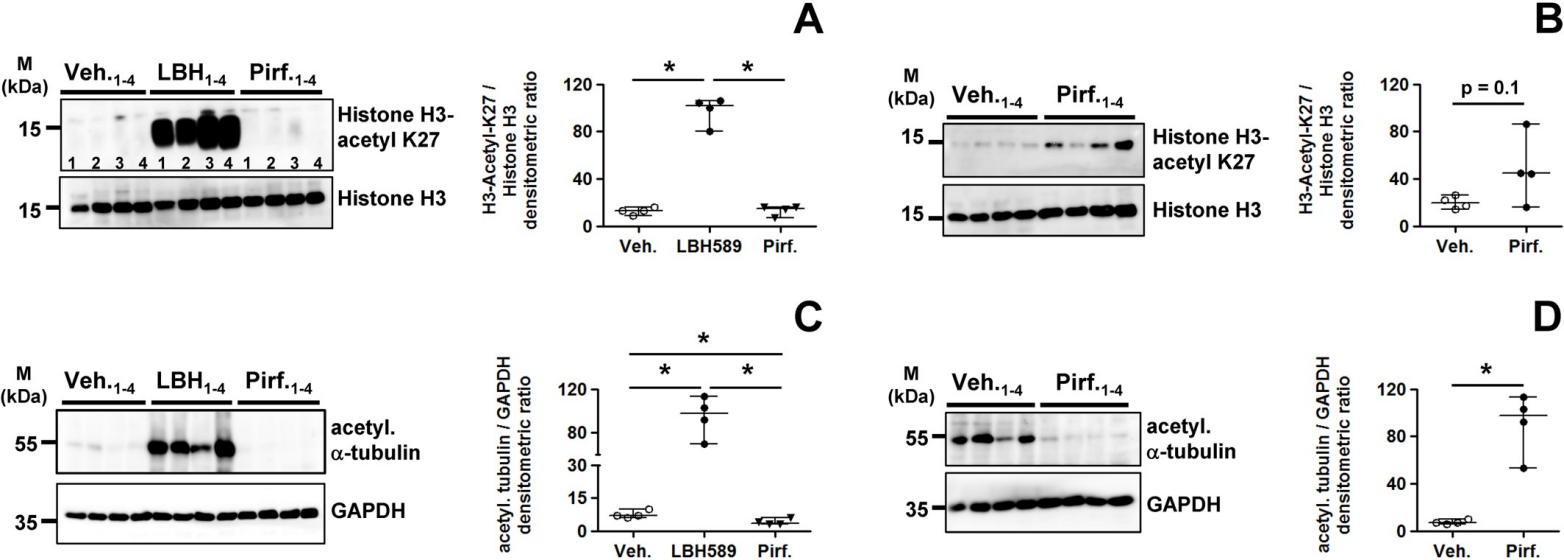
Acetylation status of the chromatin and α-tubulin in lung fibroblasts of patients with idiopathic pulmonary fibrosis in response to treatment with LBH589 or pirfenidone. Primary IPF-fibroblasts (n = 4) were incubated for 24h with vehicle [Veh., 0.25% (v/v) DMSO], panobinostat (LBH589, 85 nM) or pirfenidone (Pirf., 2.7 mM), followed by harvesting of cells. Acetylation status of histone H3 (A, B) and α-tubulin (C, D) was analyzed by quantitative immunoblotting. In dependency of research target, histone H3 or GAPDH served as loading control. (A) Histone H3-acetyl K27 (antibody-dilution 1:15000), (B) Histone H3-acetyl K27 (antibody-dilution 1:2000), (C) acetylated α-tubulin (antibody-dilution 1:60000), (D) acetylated α-tubulin (antibody-dilution 1:2000). Data are presented as median ± range of the individual values of different treatments. *p<0.05, by Mann Whitney test.

We examined the therapeutic efficacy of the HDAC inhibitor panobinostat (LBH589) and pirfenidone head-to-head in cultured primary IPF-fibroblasts (n = 4), and incubated them for 24h with vehicle [0.25% (v/v) DMSO], LBH589 (85 nM) or pirfenidone (2.7 mM, ~ 0.52 mg/mL). As control experiment for effective HDAC inhibition in response to LBH589, we first examined the acetylation status of histone H3-K27 (H3K27Ac) in all treatments. As expected, LBH589-treated IPF-fibroblasts revealed a very strong acetylation of this core histone, when compared to vehicle and pirfenidone (Fig 1A). Because we suggested an escape of detection of H3K27Ac levels in these both conditions, we repeated the immunoblot with a higher concentration of the primary antibody by using only fibroblast-lysates of vehicle- and pirfendone-treatments, and omitting the LBH589-lysates. As shown in Fig 1B, pirfenidone led also to a significant increase of H3K27-acetylation in IPF-fibroblasts, when compared to vehicle.

As LBH589 blocks as a pan-HDAC inhibitor all class-I, -II and -IV HDAC enzymes, we also assessed the acetylation status of α-tubulin, which is a substrate of the class-IIb deacetylase HDAC6 [43]. In line with HDAC6 inhibition, LBH589 resulted in a very strong increase of α-tubulin-acetylation in IPF-fibroblasts, as compared to vehicle and pirfenidone (Fig 1C). Again, a clear evaluation of α-tubulin-acetylation status in vehicle- versus pirfenidone-treated IPF-fibroblasts could not be made, and an additional immunoblot of only vehicle- and pirfenidone-treated IPF-fibroblasts was performed, by using a higher concentration of the anti-acetylated α-tubulin antibody. As shown in Fig 1D, this approach revealed basal levels of acetylated α-tubulin in vehicle-, but significantly diminished α-tubulin-acetylation in pirfenidone-treated IPF-fibroblasts.

Taken together, histone- and α-tubulin-hyperacetylation in response to LBH589 confirmed the efficacy of HDAC suppression. Importantly, pirfenidone-treatment led also to a significant increase in chromatin-acetylation, but decreased α-tubulin-acetylation in cultured IPF-fibroblasts, as compared to vehicle-treatment.

### Altered proliferation in IPF-fibroblasts in response to panobinostat- and pirfenidone-exposure

**Fig 2:**
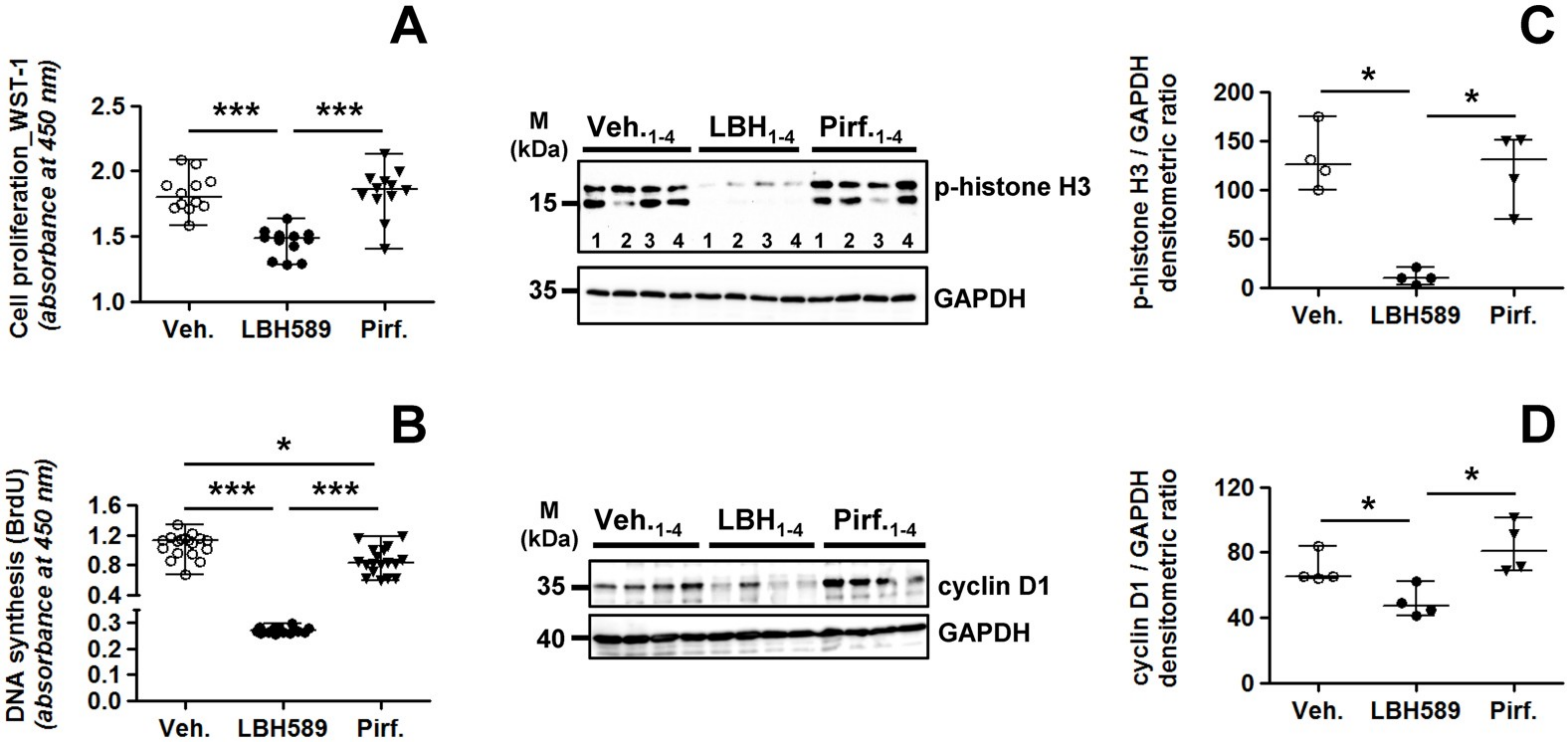
Status of proliferation in lung fibroblasts of patients with idiopathic pulmonary fibrosis in response to treatment with LBH589 or pirfenidone. Primary IPF-fibroblasts (n = 4) were incubated for 24h with vehicle [Veh., 0.25% (v/v) DMSO], panobinostat (LBH589, 85 nM) or pirfenidone (Pirf., 2.7 mM), followed by assessment of cell proliferation. (A) Proliferation of IPF-fibroblasts (n = 4) as assessed by WST-1 assay, in triplicate determination. (B) Proliferation of IPF-fibroblasts (n = 6) as assessed by BrdU incorporation, in triplicate determination. (C, D) Representative and quantitative immunoblotting for (C) p-histone H3 and (D) cyclin D1. GAPDH served as loading control. Data are presented as median ± range of the individual values of different treatments. *p<0.05, ***p<0.01, by Mann Whitney test.

We next analyzed the proliferation status in vehicle-, LBH589- and pirfenidone-treated IPF-fibroblasts. As expected, LBH589-incubation resulted in significant suppressed proliferation of IPF-fibroblasts in comparison to vehicle and pirfenidone, as assessed by WST-1 (Fig 2A) and BrdU proliferation assay (Fig 2B). Moreover and in contrast to some published reports [41,42,44–46], WST-1 and BrdU assay indicated no pronounced impairment of proliferation in IPF-fibroblasts in response to pirfenidone (Fig 2A and 2B). In addition, cell viability of pirfenidone-treated IPF-fibroblasts as reflected by mitochondrial dehydrogenase activity in WST-1 assay, did not differ from vehicle-treatment (Fig 2A).

These results were further supported by immunoblots for the proliferation markers phospho (p)-histone H3 (Fig 2C) and cyclin D1 (Fig 2D), indicating equal expression of both proteins in pirfenidone- and vehicle-treated IPF-fibroblasts, but significant suppressed levels in LBH589-treated cells.

### Altered transcription of profibrotic, ER stress- and apoptosis-related genes in IPF-fibroblasts after panobinostat- and pirfenidone-treatment

**Fig 3:**
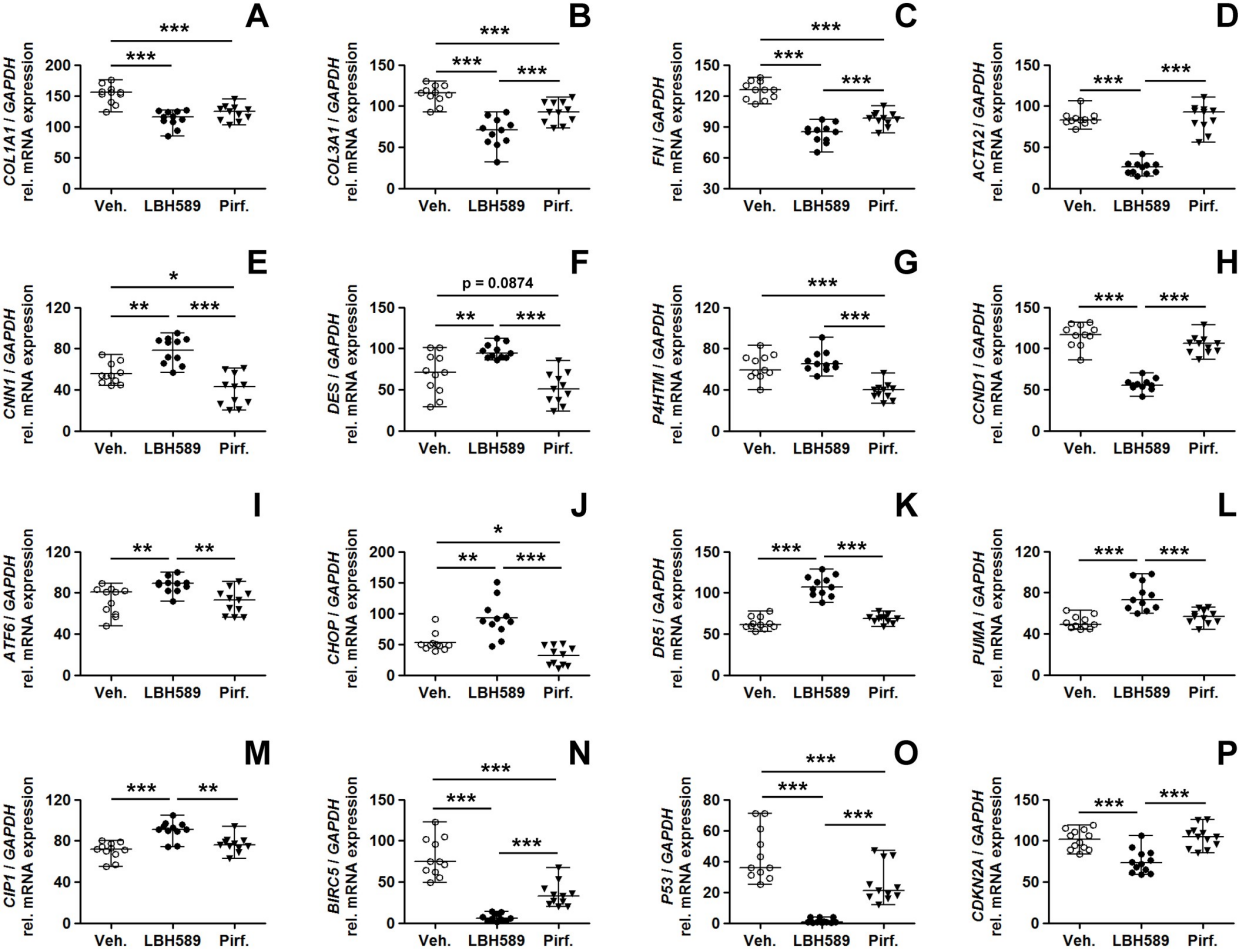
Cellular consequences in primary IPF-fibroblasts in response to treatment with panobinostat or pirfenidone. Primary IPF-fibroblasts (n = 5,6) were incubated for 24h with vehicle [Veh., 0.25% (v/v) DMSO], panobinostat (LBH589, 85 nM) or pirfenidone (Pirf., 2.7 mM). The effects of vehicle-, LBH589- and pirfenidone-treatment were analyzed by reverse transcription-polymerase chain reaction (RT-PCR) for indicated genes. (A) *COL1A1*, (B) *COL3A1*, (C) *FN*, (D) *ACTA2*, (E) *CNN1*, (F) *DES*, (G) *P4HTM*, (H) *CCND1*, (I) *ATF6*, (J) *CHOP*, (K) *DR5*, (L) *PUMA*, (M) *CIP1*, (N) *BIRC5*, (O) *P53* and (P) *CDKN2A*. Each PCR reaction was performed with 100 ng reverse-transcribed complementary DNA, followed by electrophoresis through a 2% (w/v) agarose gel containing ethidium bromide. Scanned agarose gels of indicated genes are shown in S1 Fig. Band intensities of PCR products were densitometrically quantified, and mRNA expression of indicated genes was normalized to the expression of *GAPDH*. Data are presented as median ± range of the individual values, from two independent experiments. *p<0.05, **p<0.01, ***p<0.001, by Mann Whitney test.

We then compared the effects of LBH589-, pirfenidone- and vehicle-treatment on expression of genes associated with fibrogenesis, cell survival, apoptosis and ER stress. As shown by RT-PCR in Fig 3 (and S1 Fig), pirfenidone-treatment resulted in a significant decrease in *COL1A1*- (α-1 type-I collagen, Fig 3A), *COL3A1*- (α-1 type-III collagen, Fig 3B), and *FN* (fibronectin) gene expression (Fig 3C). This was also observed to similar extent in LBH589-treated IPF-fibroblasts. Interestingly, despite effective downregulation of *COL1A1*, *COL3A1* and *FN*, the expression of *ACTA2* (α-SMA) was not observed to be reduced in response to pirfenidone as compared to vehicle, but was significantly suppressed in LBH589-treated IPF-fibroblasts (Fig 3D). Importantly, unlike LBH589-treatment, only pirfenidone resulted in a strong reduction in the mRNA expression of the profibrotic genes calponin-1 (*CNN1*, Fig 3E), desmin (*DES*, Fig 3F) and transmembrane prolyl 4-hydroxylase (*P4HTM*, Fig 3G) in comparison to vehicle-control. In line with western blot results (Fig 2D), gene expression of the proliferation marker cyclin D1 (*CCND1*) was not decreased in response to pirfenidone, but significantly downregulated in LBH589-treated IPF-fibroblasts (Fig 3H). Another observation was that, while LBH589-treatment was associated with the induction of ER stress- and apoptosis-related genes in IPF-fibroblasts, as shown by increases in *ATF6*- (activating transcription factor-6, Fig 3I), *CHOP*- (C/EBP homologous protein, Fig 3J), *DR5*- (death receptor 5, Fig 3K), *PUMA*- (p53 upregulated modulator of apoptosis, Fig 3L) and *CIP1*/p21 transcripts (Fig 3M), treatment with pirfenidone did not elicit this kind of response. Surprisingly, not only LBH589, but also pirfenidone efficiently downregulated the cancer-associated, anti-apoptosis gene *BIRC5* (survivin) in IPF-fibroblasts as compared to vehicle-treatment (Fig 3N). The suppressing effect of both drugs on *BIRC5* expression was also observed in treated IPF-fibroblast cell line CCL-134 and human embryonic WI-38 fibroblasts (S2 Fig). Finally, pirfenidone led to significant downregulation of basal *P53* gene expression in IPF-fibroblasts, but which was, as previously reported [31], paradoxically also observed in LBH589-treated cells (Fig 3O), despite upregulation of the p53 target genes *CIP1* and *PUMA*. We do not have a plausible explanation for down-regulation of *P53* mRNA expression in response to LBH589. Nevertheless, the HDAC-inhibitor-mediated reduction of *P53* does not disagree with the reported pro-apoptotic effects of LBH589, as it can trigger ER stress-induced apoptosis independently of the p53 status, as demonstrated in p53-deficient Hep3B tumor cells [47]. In agreement, p53-independent activation of *PUMA* and *CIP1* has been reported in response to HDAC-inhibitor-treatment, mediated through ER stress-induced apoptosis or histone modifications [31,48,49].

Interestingly, transcript level of the senescence-associated tumor-suppressor *CDKN2A* (p16), which were similar to *P53* found at basal levels in IPF-fibroblasts, were reduced in reponse to panobinostat-, but not pirfenidone-treatment as compared to vehicle (Fig 3P).

### Altered expression profiles of HDAC enzymes in response to panobinostat- or pirfenidone-treatment

**Fig 4:**
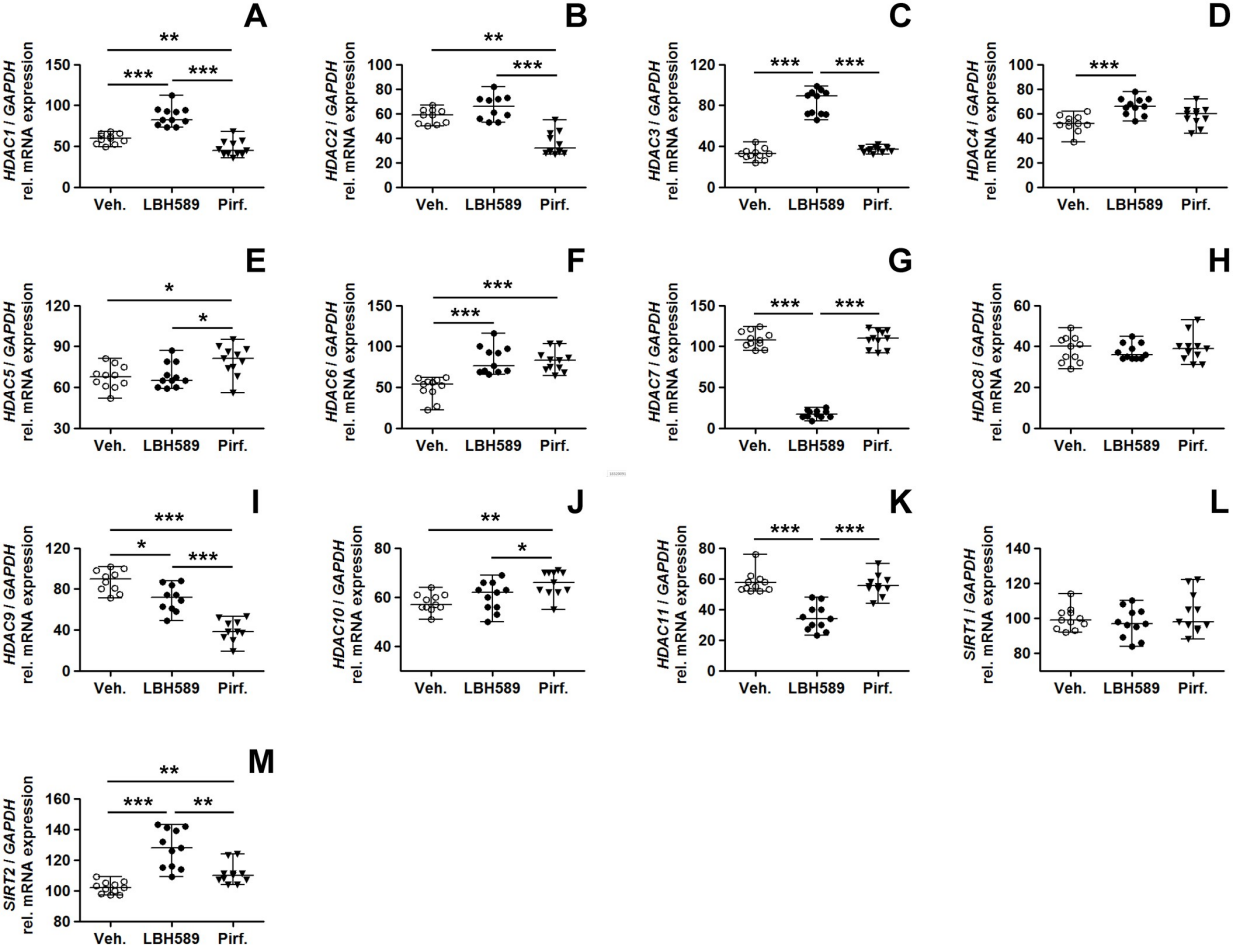
Effects of LBH589- or pirfenidone treatment on histone deacetylase gene expression in primary IPF-fibroblasts. Primary IPF-fibroblasts (n = 5,6) were incubated for 24h with vehicle [Veh., 0.25% (v/v) DMSO], panobinostat (LBH589, 85 nM) or pirfenidone (Pirf., 2.7 mM). The effects of vehicle-, LBH589- and pirfenidone-treatment were analyzed by reverse transcription-polymerase chain reaction (RT-PCR) for indicated HDAC genes. (A) *HDAC1*, (B) *HDAC2*, (C) *HDAC3*, (D) *HDAC4*, (E) *HDAC5*, (F) *HDAC6*, (G) *HDAC7*, (H) *HDAC8*, (I) *HDAC9*, (J) *HDAC10*, (K) *HDAC11*, (L) *SIRT1* and (M) *SIRT2*. Each PCR reaction was performed with 100 ng reverse-transcribed complementary DNA, followed by electrophoresis through a 2% (w/v) agarose gel containing ethidium bromide. Scanned agarose gels of indicated genes are shown in S3 Fig. Band intensities of PCR products were densitometrically quantified, and mRNA expression of indicated genes was normalized to the expression of *GAPDH*. Data are presented as median ± range of the individual values, from two independent experiments. *p<0.05, **p<0.01, ***p<0.001, by Mann Whitney test.

We compared the gene expression of HDAC enzymes in IPF-fibroblasts in response to pirfenidone-, LBH589- and vehicle-incubation (Fig 4 and S3 Fig). Compared to vehicle- and pirfenidone-treated cells, LBH589-treatment resulted in reduced *HDAC11* expression (Fig 4K) and to marked suppression of *HDAC7* (Fig 4G), paralleled by significant increases in *HDAC3*- (Fig 4C), *HDAC4*- (Fig 4D), *HDAC6*- (Fig 4F) and *SIRT2* (class-III HDAC enzyme sirtuin-2) transcripts (Fig 4M), presumably induced as compensatory mechanism due to global HDAC inhibition. These effects of LBH589 on HDAC gene activation were very similar to those observed in a previous study [31], and were thus evidently reproduced in this study. Importantly, with regard to pirfenidone-treatment, it decreased significantly the mRNAs for *HDAC1* (Fig 4A), *HDAC2* (Fig 4B) and *HDAC9* (Fig 4I, S3 Fig), and it increased the mRNAs for *HDAC5* (Fig 4E), *HDAC6* (Fig 4F), *HDAC10* (Fig 4J), and *SIRT2* (Fig 4M), as compared to LBH589- and vehicle-treatment.

**Fig 5:**
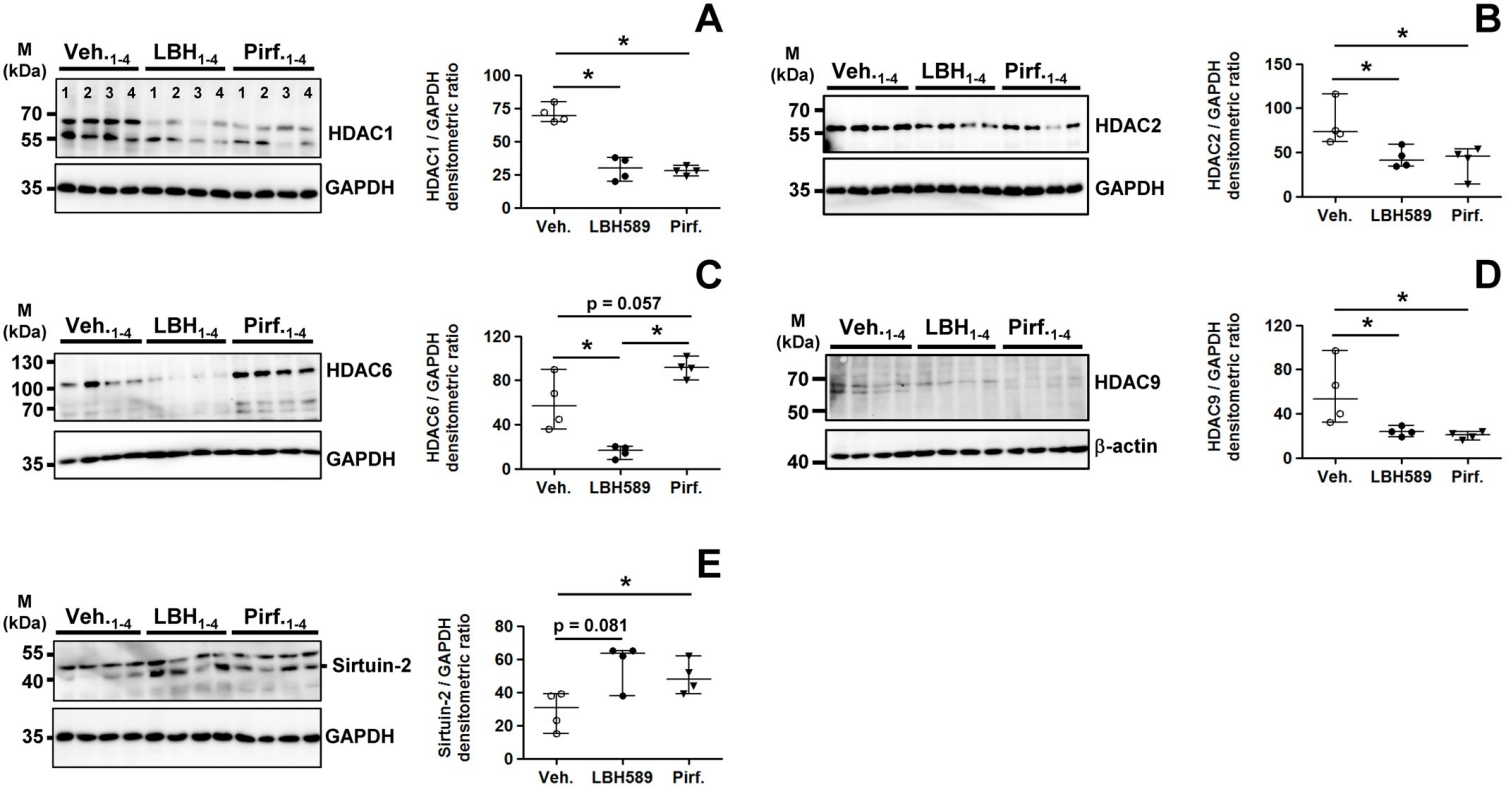
Analysis of HDAC protein expression status in primary IPF-fibroblasts in response to treatment with panobinostat or pirfenidone. Primary IPF-fibroblasts (n = 4) were incubated for 24h with vehicle [Veh., 0.25% (v/v) DMSO], panobinostat (LBH589, 85 nM) or pirfenidone (Pirf., 2.7 mM), followed by immunoblot analyses of harvested cells. (A-E) Representative and quantitative immunoblotting for (A) HDAC1, (B) HDAC2, (C) HDAC6, (D) HDAC9, and (E) sirtuin-2. GAPDH or β-actin served as loading control. Data are presented as median ± range of the individual values of different treatments. *p<0.05, by Mann Whitney test.

Inspired from the mRNA data, we then analyzed the protein expression of some differently transcribed HDAC enzymes in response to treatment with both drugs. Pirfenidone, but also LBH589, reduced significantly the protein expression of HDAC1 (Fig 5A), HDAC2 (Fig 5B), and of a ~66 kDa isoform of class-IIa-HDAC9 (Fig 5D), a HDAC comprising several alternatively spliced isoforms with profibrotic function in liver fibrosis [50]. The reduction of *HDAC9* in response to pirfenidone was already evident on mRNA level in primary IPF-fibroblasts (Fig 4I), but also in ATCC lung fibroblast cell lines CCL-134 and WI-38 (S2 Fig).

In accordance with mRNA expression, pirfenidone-treatment was associated with increased protein expression of HDAC6 (Fig 5C) and sirtuin-2 (Fig 5E). Moreover, the upregulation of both α-tubulin-deacetylases HDAC6 and sirtuin-2 [51] was in line with concomitant reduced tubulin-acetylation in pirfenidone-treated IPF-fibroblasts (Fig 1D).

### Inactivation of STAT3 signaling and fibrogenesis in IPF-fibroblasts after treatment with panobinostat or pirfenidone

**Fig 6:**
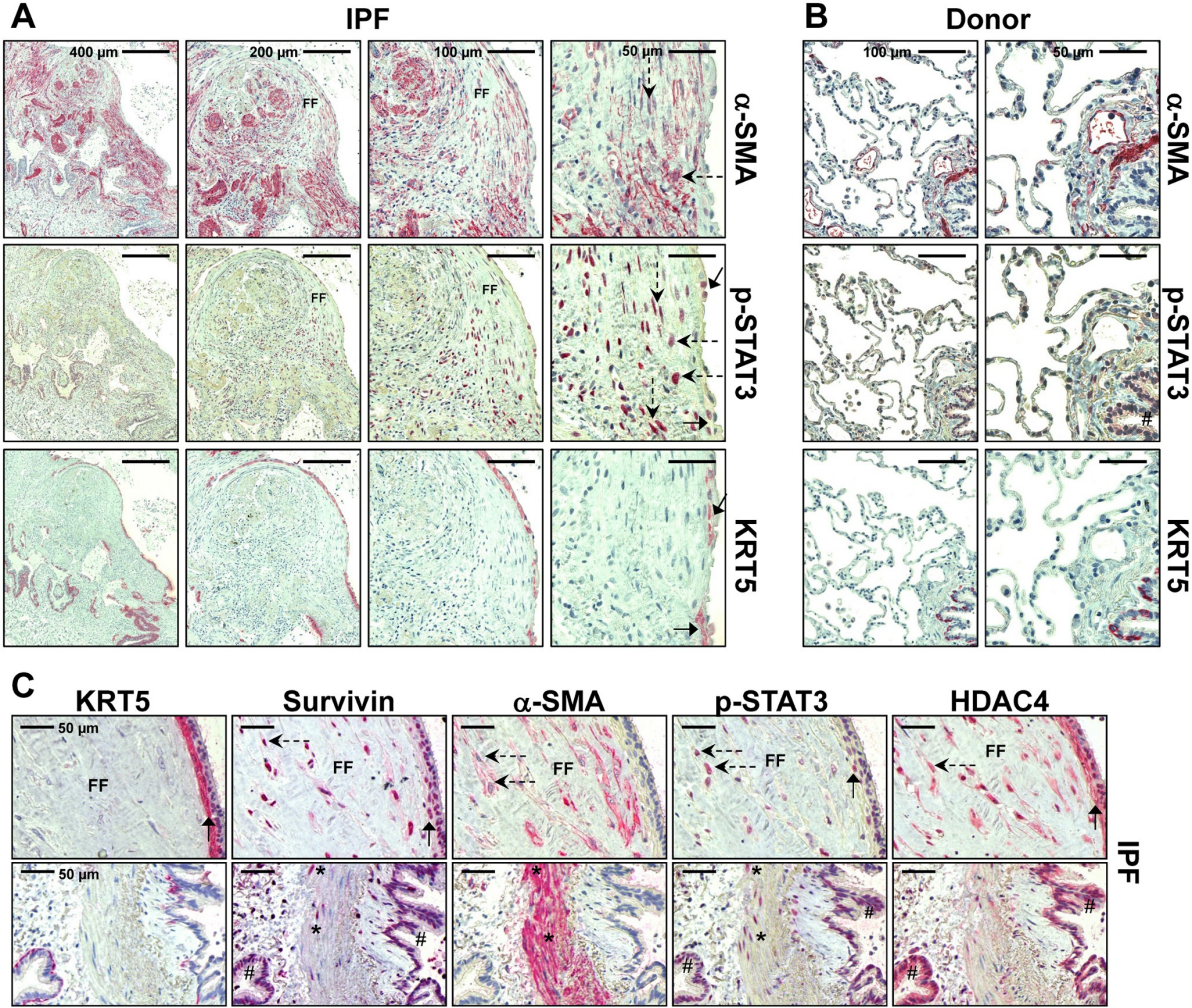
Localization of activated, phosphorylated STAT3 in fibroblast foci and overlying bronchiolar epithelium in idiopathic pulmonary fibrosis (IPF) lungs. Representative immunohistochemistry for α-SMA, phosphorylated (p)-STAT3 (Y705) and cytokeratin-5 (KRT5) in (A) IPF- and (B) normal donor lung tissue. (C) Representative immunohistochemistry for KRT5, survivin, α-SMA, p-STAT3, and HDAC4 in serial sections of IPF-lung tissue. (A) In IPF, the antibody for p-STAT3 revealed nuclear staining in myofibroblasts of fibroblast foci (FF, indicated by dashed arrows and α-SMA-staining) as well as in abnormal bronchiolar basal cells overlying FF (indicated by arrows and KRT5 staining). (B) Normal donor lungs indicated no or minimal staining in the interstitium as well as alveolar epithelium. Bronchial epithelium (indicated by hashmark) showed only faint immunoreactivity for p-STAT3. (C) Induction of p-STAT3 is observed in fibroblast foci (indicated by dashed arrows) and overlying abnormal bronchiolar epithelium (indicated by arrows and KRT5 expression), as well as in bronchioles (indicated by hashmark) in IPF, and coincided with survivin and HDAC4 overexpression in these areas. Smooth muscle cells of IPF lungs (indicated by asterisk) also revealed nuclear p-STAT3 and survivin induction.

Next, we examined the effect of LBH589 and pirfenidone on activation and phosphorylation of STAT3 in IPF-fibroblasts, in comparison to vehicle. Expression of phosphorylated STAT3 (p-STAT3-Y705), a profibrotic transcription factor, was clearly observed in the nuclei of myofibroblasts in fibroblast foci (dashed arrows in Fig 6A and 6C) as well as in overlying, abnormal bronchiolar basal cells (arrows in Fig 6A and 6C, S4 and S5 Figs). In contrast, expression of p-STAT3 was absent in interstitium of normal donor lungs, and faint in bronchial and alveolar epithelium of organ donors (Fig 6B, S4 Fig). Of note, nuclear p-STAT3 induction in myofibroblasts (dashed arrows) as well as bronchial epithelium (arrows and hashmark) was accompanied by survivin and HDAC4 expression in IPF lungs (Fig 6C and S5 Fig).

**Fig 7:**
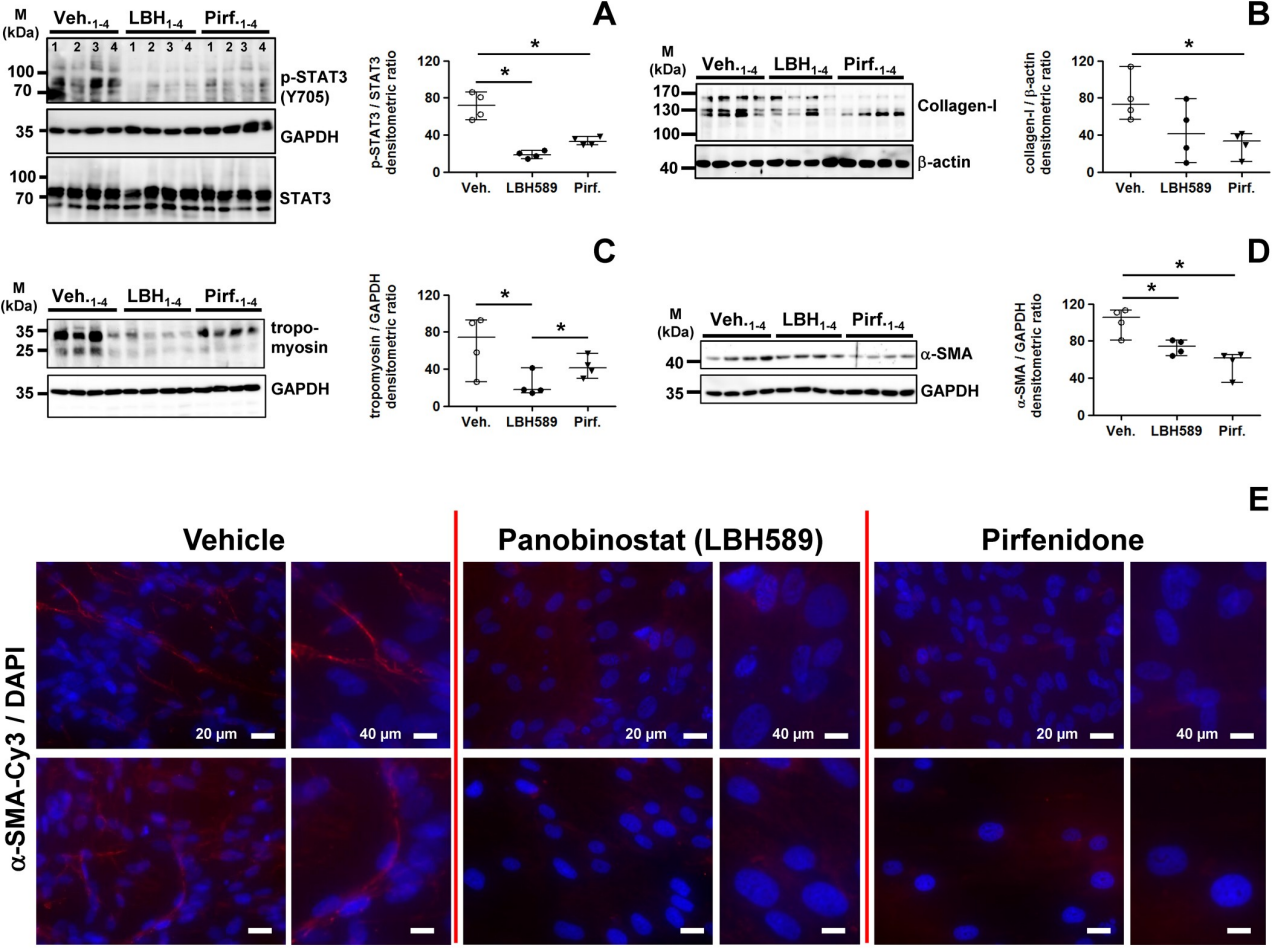
Analysis of STAT3 phosphorylation status and profibrotic protein expression in primary IPF-fibroblasts in response to treatment with panobinostat or pirfenidone. Primary IPF-fibroblasts (n = 4) were incubated for 24h with vehicle [Veh., 0.25% (v/v) DMSO], panobinostat (LBH589, 85 nM) or pirfenidone (Pirf., 2.7 mM), followed by immunoblot analyses of harvested cells. (A-D) Representative and quantitative immunoblotting for (A) p-STAT3/STAT3, (B) collagen-I, (C) tropomyosin and (D) α-SMA. STAT3, GAPDH or β-actin served as loading control. Data are presented as median ± range of the individual values of different treatments. *p<0.05, by Mann Whitney test. (E) Immunofluorescence for α-SMA-Cy3 (red) on fixed IPF-fibroblasts (n = 3) after vehicle- (left panel), LBH589- (middle panel) or pirfenidone-treatment (right panel). Nuclei were counterstained with DAPI (blue). Representative photographs are shown.

As shown by immunoblotting, LBH589 as well as pirfenidone-treatment resulted in significant reduction of profibrotic STAT3-phosphorylation in primary IPF-fibroblasts, when compared to vehicle-treated cells (Fig 7A). In accordance, both drugs also achieved reduction of collagen-I-biosynthesis, but which was eminently more affected in pirfenidone-treated cells (Fig 7B). Expression of tropomyosin, a *coiled coil* protein involved in regulation of smooth muscle contraction, was significantly diminished in LBH589-, but also virtually decreased in pirfenidone-treated IPF-fibroblasts, as compared to vehicle (Fig 7C). Importantly, protein expression of the myofibroblast marker α-SMA could be significantly reduced in IPF-fibroblasts after treatment with LBH589 or pirfenidone, in comparison to vehicle (Fig 7D). Both drugs were shown to diminish α-SMA protein also in ATCC lung fibroblastic lines CCL-134 and WI-38 (S6 Fig). In line with these data, α-SMA stress fiber formation was abrogated in LBH589- and pirfenidone-treated IPF-fibroblasts in comparison to vehicle, as shown by immunofluorescence on fixed cells (Fig 7E).

Interestingly, fluorescent phalloidin-staining for detection of F-actin structures indicated in LBH589-treated IPF-fibroblasts prominent stress fiber formation in direction to a F-actin based cell expansion, resulting in a pronounced larger cell area and increased cell speading of single fibroblastic cells (S7 Fig, middle panel), as compared to vehicle-treatments, indicating beside ‘fibrotic-based’ cell extension also linear F-actin structures (S7 Fig, left panel). We suggest, that the expansion of IPF-fibroblasts can be attributed to increased α-tubulin-acetylation after LBH589-treatment. In line with our results, the pan-HDAC-inhibitors vorinostat and trichostatin A have been previously shown to stretch and expand cancer cells, and it is suggested that such changes in cytoskeletal dynamics are associated with decreased motility and required for apoptotic cell death [52–54]. Importantly, cell extension was impaired and abrogated in response to pirfenidone-treatment, thereby indicating predominantly linear F-actin structures (S7 Fig, right panel).

### Panobinostat, but not pirfenidone, induced ER stress and apoptosis in IPF-fibroblasts

**Fig 8:**
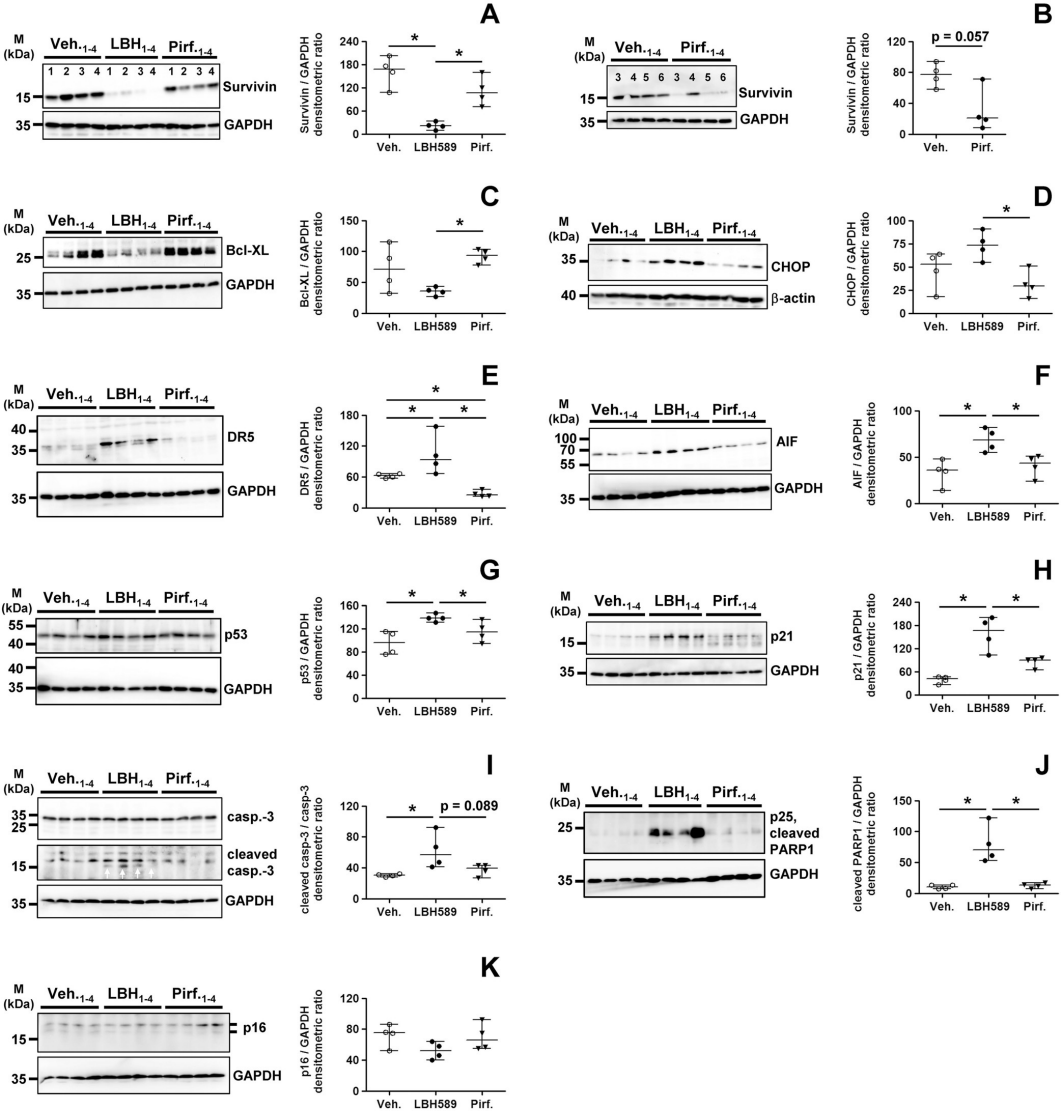
Analysis of pro-apoptotic signaling in primary IPF-fibroblasts in response to treatment with panobinostat or pirfenidone. Primary IPF-fibroblasts (n = 4) were incubated for 24h with vehicle [Veh., 0.25% (v/v) DMSO], panobinostat (LBH589, 85 nM) or pirfenidone (Pirf., 2.7 mM), followed by immunoblot analyses of harvested cells. (A-K) Representative and quantitative immunoblotting for (A, B) survivin, (C) Bcl-XL, (D) CHOP, (E) DR5, (F) AIF, (G) p53, (H) p21, (I) caspase-3/cleaved caspase-3, (J) cleaved PARP1-p25, and (K) p16. GAPDH or β-actin served as loading control. For survivin-immunoblot (B), additional IPF-fibroblasts (patients 5 and 6) were analyzed. Data are presented as median ± range of the individual values of different treatments. *p<0.05, by Mann Whitney test.

Finally, we researched apoptotic signaling in IPF-fibroblasts after treatment with LBH589 or pirfenidone versus vehicle. In line with the mRNA data, LBH589 and pirfenidone effectively decreased protein expression of survivin as compared to vehicle (Fig 8A and 8B), but suppression of this cancer-specific protein was more effective in response to the strong pan-HDAC inhibitor LBH589 (Fig 8A). Importantly, the anti-apoptosis gene Bcl-XL could not be down-regulated by pirfenidone-, but significantly suppressed by LBH589-treatment in IPF-fibroblasts (Fig 8C). Again, an obvious observation was, that pirfenidone-treatment was not associated with an induction of pro-apoptotic signaling, as compared to vehicle, whereas such was easily observed in response to LBH589-treatment of IPF-fibroblasts, as evident by the induction of the ER stress-factor CHOP (Fig 8D), the CHOP-target gene DR5 [55] (Fig 8E) and the mitochondrial apoptosis-inducing factor (AIF) (Fig 8F), which was paralleled by p53 (Fig 8G) and p21 upregulation (Fig 8H) as well as enhanced caspase-3- (Fig 8I) and PARP1-cleavage (Fig 8J). Although the LBH589 mediated p53-upregulation is not reflected by the mRNA data (Fig 3O), we suggest that its enhanced protein level is due to increased acetylation and consequent stabilization of p53. Interestingly, protein level of the senescence-associated tumor suppressor p16, which is like p53 expressed at basal levels in IPF-fibroblasts, were not altered in response to LBH589- or pirfenidone-treatment (Fig 8K).

**Fig 9:**
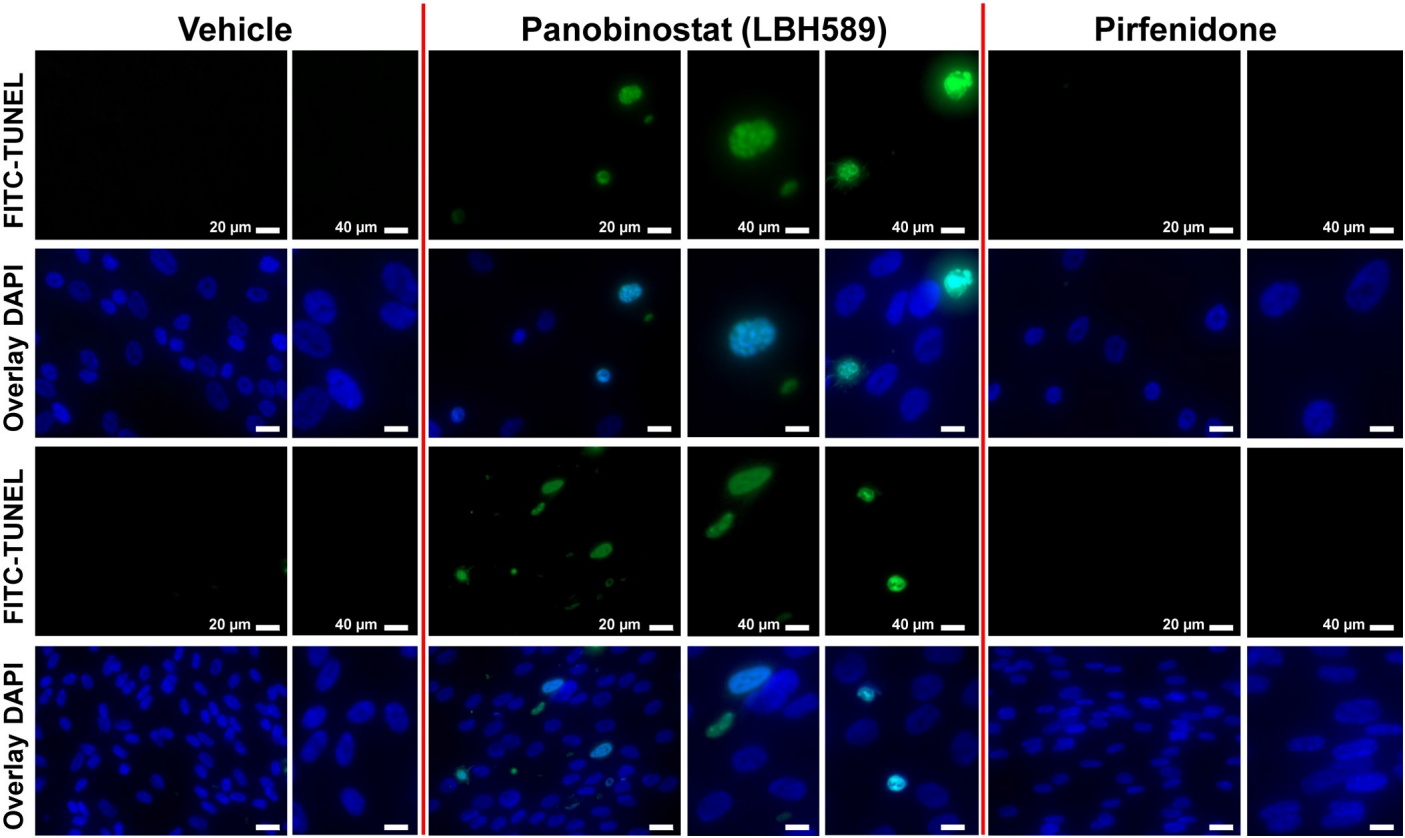
Induction of apoptosis in primary IPF-fibroblasts in response to treatment with panobinostat measured by TUNEL assay. Primary IPF-fibroblasts (n = 4) were incubated for 24h with vehicle [Veh., 0.25% (v/v) DMSO], panobinostat (LBH589, 85 nM) or pirfenidone (Pirf., 2.7 mM), followed by immediate assessment of *in situ* cell death using TUNEL method. Fluorescence microscopy indicates representative photographs of TUNEL-positive staining of apoptotic nuclei (FITC, green), and of DAPI-counterstained apoptotic nuclei (bright blue) and non-apoptotic nuclei (dark blue) of panobinostat-treated IPF-fibroblasts in overlay images (middle panel). TUNEL-positive nuclei indicated apoptotic bodies containing dense nuclear fragments involving chromatin condensation. No signs of significant apoptosis was observed in vehicle- (left panel) and pirfenidone-treated IPF-fibroblasts (right panel). Results are representative of two independent experiments.

In summary, these data now suggest, that beside p53-dependent apoptosis, ER stress-induced apoptosis mediated by CHOP, which ultimately leads to activation of the mitochondrial apoptotic pathway, plays a key role in LBH589-mediated inactivation of IPF-fibroblasts. We also performed TUNEL assay in fixed IPF-fibroblasts after 24h-exposure to vehicle, LBH589 and pirfenidone, and observed TUNEL-positive nuclei with apoptotic bodies containing dense nuclear fragments only in LBH589-, but not vehicle- or pirfenidone-treated IPF-fibroblasts (Fig 9).

Taken together, the head-to-head comparison of LBH589 versus pirfenidone clearly indicated, that LBH589 downregulates profibrotic gene expression while increasing apoptosis, whereas pirfenidone-treatment maintained fibroblast-survival by effective suppression of pro-fibrotic and cancer-like phenotypes.

## Discussion

Several clinical trials at present have shown that pirfenidone and nintedanib slow the decline of lung function in IPF-patients, and reduce the rate of disease progression by 50% [3,4,56–58]. Pirfenidone, in addition to its inhibitory effects on the reduction of forced vital capacity (FVC) [3,57], has also been shown to have a significant effect on the mortality of the IPF-patients [58]. Although considered as an anti-fibrotic agent, its exact mechanism of action is unknown. The most commonly reported adverse effects of pirfenidone were gastrointestinal symptoms (nausea, dyspepsia, vomiting, anorexia) and skin related (rash and photosensitivity); but these effects were well tolerated in patients participating in clinical trials, especially when the dose was decreased [3,58]. However, a curative therapy for IPF is still lacking, which emphasizes the need for all kinds of research.

IPF has a series of biological abnormalities (genetic and epigenetic alterations) and risk factors (aging, smoking and environmental exposures) in common with cancer [59]. Results from our group and others suggest that specifically epigenetic mechanisms and histone modifications account for the aggressive phenotype of fibrotic fibroblasts, which indicated ‘cancer-like’ upregulation of various HDAC enzymes, and that HDAC inhibitors may offer a new therapeutic strategy in IPF by increasing myofibroblast susceptibility to apoptosis and blocking fibrotic remodelling [31–33,60]. HDAC inhibitors have been reported a long time as successful anticancer agents as they induce cell cycle arrest and apoptosis selectively in cancer cells by increasing the acetylation status of histones and various non-histone proteins, including transcription factors, leading to such altered gene expression profiles and cellular signaling [39]. With regard to lung fibrosis, Sanders and coworkers could show that the hydroxamic acid-based pan-HDAC inhibitor SAHA decreased lung fibrosis and improved lung function in bleomycin-treated mice [33]. Similarly, Conforti F and coworkers reported potent anti-proliferative and anti-fibrotic properties of the class-I-HDAC inhibitor romidepsin on fibrotic lung fibroblasts *in vitro* and *in vivo* [60]. Also treatment of isolated fibroblasts from IPF-patients with the pan-HDAC inhibitor panobinostat/LBH589 reduced significantly profibrotic genes and also diminished the synthesis of anti-apoptosis molecules [31]. Thus, these anticancer agents hold great promise and could be readily progressed into an IPF clinical trial, as SAHA (vorinostat) and romidepsin are licensed and established therapies for cutaneous T-cell lymphoma (CTCL) [39], and LBH589 for multiple myeloma [34–36]. LBH589 is an orally available, novel hydroxamic acid pan-HDAC inhibitor that potently inhibits all class I, -II and IV HDAC enzymes at low nanomolar concentrations [34,38,39], and is reported to be at least 10-fold more potent than SAHA [61]. In the present study, we compared its therapeutic effect with the IPF-drug pirfenidone head-to-head in isolated IPF-fibroblasts *in vitro*.

Surprisingly, the WST-1 and BrdU proliferation assays revealed, that pirfenidone did not greatly inhibit the proliferation of IPF-fibroblasts at 2.7 mM concentration as compared to vehicle, but which was, as expected, significantly reduced in response to the anti-cancer drug LBH589. This result was corroborated by protein expression analyses for proliferation markers p-histone H3 and cyclin D1, indicating suppression only in LBH589-incubated IPF-fibroblasts in comparison to vehicle- and pirfenidone-treatments with same expression status of both markers. In agreement, only LBH589, but not pirfendone, downregulated *CCND1* gene transcription versus vehicle.

This observation stands somewhat in contrast with some reports illustrating a significant inhibitory effect of pirfenidone on proliferation at concentrations 1.0–10.0 mM in a variety of cell types *in vitro* after 24h exposure, including mesenchymal stem cells and fibrotic fibroblasts [41,42,44–46,62]. We do not have a plausible explanation for this discrepancy, in particular when low concentrations (1–3 mM) of this IPF-drug were examined in contrasting studies [41,44–46]. As we did also not observe a pronounced impairment of proliferation in low-density cultured IPF-fibroblasts in response to 24h-treatment with 2.7 mM pirfenidone, we conclude that proliferation is not greatly affected in IPF-fibroblasts at this concentration. Importantly, the concentrations of pirfenidone used in reported *in vitro* experiments (1.0–10.0 mM) are 10 to 100 fold higher than maximum drug concentrations observed in the blood of patients with IPF (~ 0.1 mM) during standard treatment (three daily doses of 801 mg pirfenidone) [63]. We selected 2.7 mM of pirfenidone for *in vitro* experiments, because we and others did not observe any significant effects on profibrotic gene expression at concentrations lower than 2.6 mM (~ 0.5 mg/mL) [30,41].

A further important observation in this study was, that pirfenidone resulted in a slight, but significant increase of histone acetylation in cultured IPF-fibroblasts versus vehicle. In line with this, we observed a significant reduction of class I-HDACs HDAC1 and -2 and class IIa-deacetylase HDAC9 which all mainly deacetylate histones, on mRNA- as well as protein level, in response to pirfenidone as compared to vehicle-treatment. These results indicated that pirfenidone might act indirectly as an epigenetic modulator through downregulation of HDAC enzymes. As expected, LBH589-treated IPF-fibroblasts indicated much more pronounced and excessive histone-acetylation compared to vehicle- and pirfenidone, due to efficient pan-HDAC inhibition, but was also associated with a depletion of HDAC1, -2, -6 and -9 on the post-translational level (which is often observed in response to [pan-]HDAC inhibition thereby potentiating repression of HDAC activity [64]), while transcript levels of *HDAC1* and *HDAC2* were slightly increased (presumably as compensatory mechanism).

Furthermore, we observed that both LBH589- and pirfenidone-treatment resulted in significant suppression of STAT3-phosphorylation at Tyr705 and its activation, as well as in reduction of ECM-associated proteins α-SMA, tropomyosin and collagen-I in IPF-fibroblasts, when compared to vehicle. Notably, the inhibition of STAT3 activation in pirfenidone-treatments did not significantly differ in extent from LBH589-treated IPF-fibroblasts indicating histone-hyperacetylation. Likewise, reduction of profibrotic genes *COL1A1*, *COL3A1* and *FN* was evident on the transcriptomic level in response to both drugs, whereas only the mRNA for *ACTA2* was downregulated in LBH589- versus vehicle- and pirfenidone-treatment, suggesting that pirfenidone regulates *ACTA2*/α-SMA during translation.

Much to our surprise, pirfenidone only was capable in reducing transcription of profibrotic ECM-associated genes *CNN1* (calponin), *DES* (desmin) and *P4HTM* (transmembrane prolyl 4-hydroxylase) as compared to LBH589- and vehicle-treatment, with *CNN1* and *DES* being paradoxically even upregulated in IPF-fibroblasts in response to LBH589.

Further, the anti-apoptosis gene *BIRC5*/survivin, which is overexpressed in fibroblast foci of IPF-lungs and suspected to mediate apoptosis-resistance and persistence of myofibroblasts [65], was suppressed after LBH589-treatment, but also appeared to be partially downregulated in pirfenidone-treated IPF-fibroblasts. Taken together, these data indicate that pirfenidone can somewhat compete with the strong epigenetic modifier LBH589 in reducing expression of profibrotic and cancerous genes.

The reduction of ECM-associated genes and *BIRC5*/survivin in response to LBH589 and pirfenidone may partially be due to attenuated STAT3 activation under these conditions. STAT3 is a latent cytoplasmic transcription factor that is activated by multiple cytokines and growth factors, including TGF-β1, PDGF, as well as IL-6 family of cytokines. In response to such ligands, STAT3 becomes specifically phosphorylated at Tyr705 by growth factor/cytokine receptor-associated Janus kinases (JAK). Activated (phosphorylated) STAT3 translocates to the nucleus, where it binds with DNA and regulates gene transcription, which has been reported to be especially associated with increased proliferation as well as myofibroblast differentiation in fibroblastic cell populations [19,66,67]. Moreover, STAT3 is found to be active in many human cancers, and crucially involved in induction of *BIRC5*/survivin expression [68], and has been observed to correlate with increased invasion, metastasis and chemo-resistance in cancer [69]. Levels of p-STAT3 have been shown to be elevated in the bleomycin mouse model and in patients with IPF [19,20,67], and were recently observed to be overexpressed in the nucleus of myofibroblasts, alveolar macrophages and of AECIIs adjacent to fibroblast foci, while being absent in non-fibrotic healthy control lungs [20]. In the present study, we found nuclear p-STAT3 (Tyr705) to be exclusively expressed by myofibroblasts of fibroblast foci and (abnormal) bronchiolar basal cells, which have been suggested to initiate and propagate the bronchiolization process of distal alveoli in IPF [70]. Moreover, inhibition of the STAT3 pathway by the specific small molecule inhibitor C-188-9 has been demonstrated to decrease fibroblast-to-myofibroblast differentiation *in vitro* and development of pulmonary fibrosis in bleomycin-treated mice *in vivo* [20].

Though, the detailed molecular mechanisms underlying LBH589- and pirfenidone-mediated abrogation of STAT3-phosphorylation in isolated IPF-fibroblasts which is a novel finding of this study, remain to be elucidated. Various reports from the cancer field suspect, that increased deacetylation of STAT3 seems to be required for its phosphorylation and nuclear translocation, as various specific class I and class II-HDAC inhibitors (valproic acid, entinostat, romidepsin, ‘mercaptoacetamid-based HDACi W2’) as well as the pan-HDAC inhibitor LBH589 were shown to suppress STAT3-phosphorylation/activation in a variety of malignant cancers, and which was associated with significantly reduced migration and invasiveness of cancer cells [71–74]. Pang M and coworkers treated mice with unilateral uretal obstruction (UUO) and sham-operated mice immediately after ligation daily with the pan-HDAC inhibitor trichostatin-A (TSA) and observed that attenuation of profibrotic gene expression, myofibroblast biogenesis and interstitial fibrosis in injured kidneys through pan-HDAC inhibition was associated beside histone-hyperacetylation specifically with the abrogation of STAT3 phosphorylation at Tyr 705 in renal fibroblastic cells [66]. Because HDAC1, -2, and -3 have been reported to reduce STAT3-acetylation [75], it can be suggested that the aforementioned HDAC inhibitors increase the acetylation status of STAT3, which can be acetylated at lysines 679, 685, 707 and 709 (acetylated lysine residues in the human STAT3 protein [76]). The consequent STAT3-hyperacetylation might lead to conformational changes of the protein and prevent adjacent tyrosine phosphorylation at Tyr705 and STAT3 activation. In addition, our data suggest that pirfenidone might also be involved in STAT3 acetylation, due to its ability to reduce HDAC1 and -2 expression levels.

However, despite the reported evidence that HDAC inhibitors inhibit STAT3 activation through abolishing STAT3-phosphorylation at Tyr705 in pathological conditions such as cancer and kidney fibrosis, the exact mechanism remains elusive. Another possibility is that cross talk between phosphorylation and acetylation occurs upstream at the JAKs and/or various growth factor/cytokine receptors that regulate STAT3 activation, and which might also undergo acetylation and consequent altered enzymatic activites. In addition, altered expression profiles due to acetylation of chromatin and transcription factors may also contribute to suppressed JAK/STAT3 signaling through induction of (protein tyrosine) phosphatases, altered cellular signal transduction or reduction of cytokines/growth factors. In this regard, STAT3-phosphorylation may be also disrupted indirectly upon HDAC inhibition.

The same can be in part suggested for pirfenidone, since this drug is reported to reduce the synthesis of various profibrotic cytokines and growth factors, including TGF-β1 and PDGF, in bleomyin-induced lung fibrosis in rodents [25–29]. As some of these profibrotic ligands were reduced at the transcriptional level in response to pirfenidone [26], it can be suspected that this might be due to the indirect supportive effect of pirfenidone on chromatin-acetylation as result of class I-HDAC reduction, as observed in this study. Because pirfenidone up to 10 mM concentration has been shown to be ineffective in chelating divalent metal ions (dications), such as Fe^2+^ [77], it can be assumed that this drug cannot inhibit the deacetylase activity of Zn^2+^ dependent HDAC enzymes.

An additional novel finding of this study was, that pirfenidone led to an increase of α-tubulin-deacetylation, a surrogate marker of HDAC6 activity [43], in IPF-fibroblasts. The class-III HDAC-enzyme sirtuin-2 has also been reported to deacetylate α-tubulin [51]. Accordingly, HDAC6 and sirtuin-2 were upregulated on mRNA and protein level in response to pirfenidone, as compared to vehicle. As expected, the pan-HDAC inhibitor LBH589 led to hyperacetylation of α-tubulin, and was opposite to the effect by pirfenidone. Moreover, HDAC6 overexpression has been observed in myofibroblasts within fibroblast foci of IPF-lungs, and fibroblast-isolates from IPF-patients accordingly indicated decreased acetylation of α-tubulin [31]. Increased HDAC6 expression and consecutive α-tubulin-deacetylation has also been encountered in normal lung fibroblasts in response to TGF-β1-exposure [78]. Other groups have reported that HDAC6 mediates TGF-β1-induced epithelial-mesenchymal transition (EMT) via SMAD3 activation in A549 cells, which was accompanied by α-tubulin-deacetylation and mesenchymal stress fiber formation [79,80]. Because HDAC6 is as a tubulin-deacetylase crucially involved in the aggresome pathway which functions as an alternative protein degradation mechanism in the cell through autophagic clearance of misfolded proteins [43], its presence has been suggested in part to be responsible for the resistance to proteasome inhibitors in patients with various cancers, especially in patients with relapsed and/or refractory multiple myeloma [36]. These observations lead to the suggestion that blocking α-tubulin-deacetylation through inhibition of HDAC6 deacetylase activity may have a therapeutic effect in cancers and fibrotic lung disease. Saito S and coworkers [78] observed that tubastatin, which has been widespread reported as a selective HDAC6 inhibitor, led to hyperacetylation of α-tubulin and repressed TGF-β1-induced expression of type-I collagen in lung fibroblasts, through inducing dephosphorylation of Akt and subsequent repression of the HIF-1α-VEGF axis. Moreover, tubastatin also ameliorated bleomycin-induced lung fibrosis in mice *in vivo*, whereas *Hdac6*^(-/-)^ knockout mice where not protected against collagen production and lung fibrosis induced by bleomycin, as compared to wild-type mice, despite pronounced hyperacetylation of α-tubulin in isolated lung fibroblasts of Hdac6 deficient mice. *In vitro*, siRNA mediated gene silencing of *HDAC6* in TGF-β1 treated normal human lung fibroblasts indicated significantly increased α-tubulin acetylation, but did not prevent TGF-β1 induced collagen-I (*COL1A1*) production. These results unequivoally suggested that reduction of collagen-I in response to the ‘selective’ HDAC6 deacetylase inhibitor tubastatin was not due to increased acetylation of α-tubulin, and suggested considerable off-target effects of this inhibitor aside from HDAC6 which remain to be identified [78]. Moreover, in our study, pirfenidone prevented production of collagen-I and other profibrotic molecules in the presence of increased HDAC6 and concomitant significant reduction of α-tubulin acetylation in treated IPF-fibroblasts, thus clearly indicating that targeting α-tubulin deacetylation does not affect myofibroblast differentiation. Because α-tubulin deacetylation is involved in aggresome mediated autophagy of ‘unwanted’ misfolded proteins during increased ER stress thereby significantly contributing to cell survival [43], we consider its upregulation in response to pirfenidone, however, as a survival mechansim in IPF-fibroblasts, despite the established favourable anti-fibrotic effects of this drug. Regarding the strong HDAC inhibitor LBH589, which induced α-tubulin hyperacetylation in treated IPF-fibroblasts, we suggest that inhibition of profibrotic signaling was mainly due to strong histone acetylation and chromatin-transcription, as well as interference with signal transduction mechanisms (such as STAT3 pathway) mediated by HDACs. It is believed that in IPF-fibroblasts the HDAC silenced genes are in part repressors of anti-apoptosis and profibrotic genes, which become transcriptionally activated upon HDAC inhibition thereby leading to suppression of such genes [31,33].

An additional novel anti-fibrotic mechanism of pirfenidone observed in this study was the marked downregulation of *HDAC9* on both transcriptional and translational levels in primary IPF-fibroblasts. Increased presence of HDAC9 has been reported to play a profibrotic role in the activation of hepatic stellate cells (HSC) during liver fibrosis *in vivo*, whereas knockdown of *HDAC9* decreased TGF-β1-induced fibrogenic gene expression in the human HSC cell line LX-2 [50]. Interestingly, we observed suppression of *HDAC9* transcription in a variety of epithelial and mesenchymal ATCC cell lines (A549, MLE-12, WI-38, CCL-134) in response to pirfenidone (partly shown), suggesting that *HDAC9* may be an universal direct target of this drug. Also LBH589 led to a significant reduction of HDAC9 expression, but to lesser extent as compared to pirfenidone.

Finally, the fundamental difference between the effects of LBH589 and pirfenidone was, that LBH589-treatment resulted in cell cycle arrest and induction of apoptosis in IPF-fibroblasts, as indicated by induction of p53/p21 and several apoptosis-inducing pathways, including survivin-suppression and ER stress-mediated apoptosis involving CHOP and the mitochondrial apoptotic pathway, whereas pirfenidone did not exert such responses. In addition, LBH589, but not pirfenidone, led to upregulation of AIF which is described to induce the release of cytochrome c and caspase-9 from mitochondria upon apoptosis [81], but also to initiate a caspase-independent pathway by causing DNA fragmentation and chromatin condensation. Moreover, caspase-independent mechanisms via AIF are in part mediated through survivin-reduction, which induces the translocation of AIF to the nucleus [82]. Further, LBH589, but not pirfenidone, suppressed Bcl-XL expression thus again contributing to mitochondrial apoptotic pathway. LBH589 led also to induction of DR5, presumably through ER stress/CHOP and/or histone modifications, and which triggers via caspase-8 activation apoptotic cell death [55]. Again, none of these pro-apoptotic factors were found to be enhanced upon pirfenidone-treatment, and TUNEL assay also indicated no signs of apoptosis in pirfenidone-treated IPF-fibroblasts. Remarkably, it has been demonstrated that even substantially higher concentrations of pirfenidone (5–10 mM) have no significant apoptotic or cytotoxic effects on fibroblastc cell populations *in vitro* [62].

Nevertheless, pirfenidone was, in part, capable in attenuating the ‘cancer-like’ phenotype of IPF-fibroblasts, through partial downregulation of the cancer-associated gene *BIRC5* (survivin). The reduction of *BIRC5* expression in response to pirfenidone (and panobinostat) can be in part surely attributed to inactivation of STAT3, but also to inhibition of prosurvival PI3K/AKT pathway [83]. Phosphorylation of AKT has widely been demonstrated to be reduced in response to pan-HDAC inhibition [84] as well as upon pirfenidone-treatment [30,45]. However, the partial reduction of survivin in response to pirfenidone did not result in induction of apoptosis, presumably due to maintenance of potent survival mechanisms such as Bcl-XL and upregulated α-tubulin-deacetylation (aggresome-mediated autophagy). In addition to apoptosis-resistance, survivin has also been implicated directly in EMT as well as dedifferentiation of non-stem cancer cells into cancer stem cells [83,85]. Thus, partial survivin-downregulation in response to pirfenidone might also indicate the induction of maintenance of “normal” differentiated fibroblastic cells and tissue, and reduction of the cancer-like phenotype of IPF-fibroblasts. In agreement, various reports exist that pirfenidone exhibits significant anti-cancer properties. It was shown that pirfenidone inhibited tumor growth in human glioma cells [86] and in panreatic cancer cells [87], as well as reversed EMT in human lung adenocarcinoma [88]. These effects were in part also due to suppressed TGF-β1 signaling in tumors as well as the tumor microenvironment in response to pirfenidone [59,88,89], and remain to be elucidated in detail. Because lung cancer is among the most crucial comorbidities in patients with IPF [59], its prevention (or therapy) is critical in patients with IPF (and IPF-patients with concomitant cancer). In line with reported anti-cancer activity of pirfendone, a very recent retrospective review of 261 IPF-patients with and without pirfenidone revealed that the incidence of lung cancer was significantly lower in patients treated with pirfenidone than in the non-pirfenidone group [90].

Although LBH589 is reported to be selectively cytotoxic towards ‘abnormal’ tumor cells whereas normal healthy cells are relatively unaffected [34,39], patients with multiple myeloma receiving LBH589 indicated significant adverse effects [35,36]. LBH589 (Farydak^®^) was recently approved in the US and EU in combination with bortezomib (BTZ) and dexamethasone (Dex), for the treatment of patients with relapsed and/or refractory multiple myeloma who have received at least two prior treatment regimens including bortezomib and an immunomodulatory drug [36]. Significant side effects (grade 3–4) associated with LBH589 presence encountered in clinical studies included diarrhea, fatigue, nausea, vomiting, thrombocytopenia, anemia, neutropenia and lymphopenia; but they were considered at the time of registration acceptable, because the addition of LBH589 to BTZ and Dex resulted in a clinically meaningful and significant improvement of progression-free survial compared with placebo plus BTX and Dex [as reported in the randomized, double-blind, phase III PANORAMA 1 (Panobinostat Oral in Multiple Myeloma 1) trial] [36,91]. Additional clinical studies are underway to determine the best way to use the drug in a more safe and effective manner in patients with relapsed/refractory multiple myeloma [36,92]. LBH589 is also currently under development and being tested (either alone or in combination with other drugs) for the treatment of other haematological malignancies (including chronic and acute myeloid leukemia, chronic lymphocytic leukemia, and myelofibrosis) and solid tumors (including colorectal cancer, neuroendocrine tumors, prostate cancer, and renal cancer), as well as AIDS [92,93]. However, although LBH589 indicated significant anti-tumor activities in a wide range of lung cancers [small cell lung cancer (SCLC) and non-small cell lung cancer (NSCLC)] *in vitro* and in animal models *in vivo* [94,95], a phase II study of LBH589 in pretreated patients with SCLC was prematurely discontinued because of lack of activity [96]. Nevertheless, modest clinical activity of LBH589 (involving tumor shrinkage in SCLC) combined with a favourable safety profile in pretreated SCLC patients was observed [96]. At present, no clinical studies of HDAC inhibitors in patients with IPF exist.

Preclinical studies of the related pan-HDAC inhibitor SAHA in the mouse model of bleomycin-induced lung fibrosis demonstrated significant attenuation of development of pulmonary fibrosis and improvement of lung function in mice [33]. These observations and the results from our *in vitro* studies on human IPF-fibroblasts encourage us to examine in the near future the therapeutic efficacy of LBH589 in rodent models of lung fibrosis and in human IPF *in vivo*.

**Fig 10:**
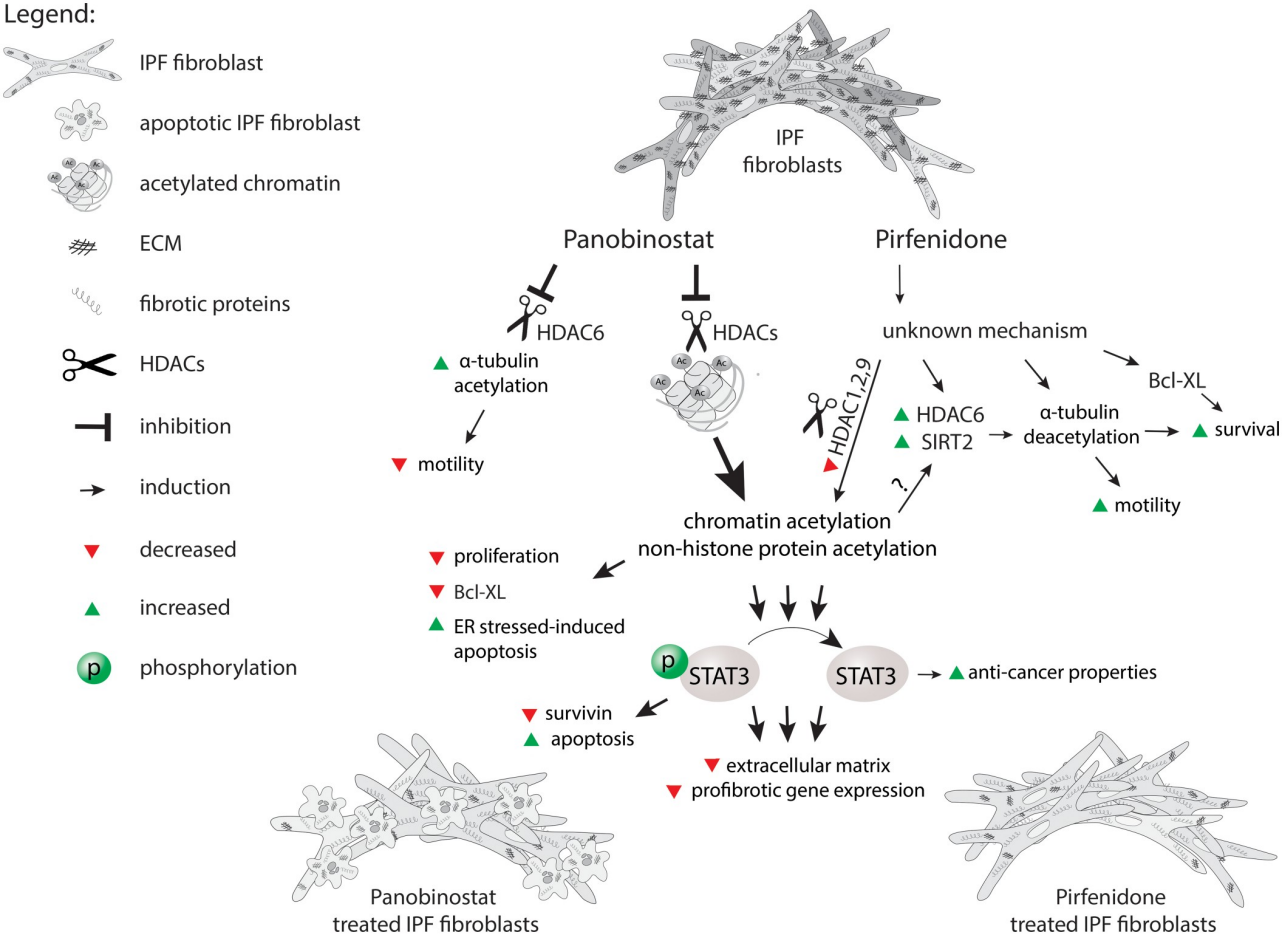
Comparison of the effects of panobinostat/LBH589 and pirfenidone on fibrotic activity and survival of primary IPF-fibroblasts *in vitro*.

Taken together, the head-to-head comparison of LBH589 versus pirfenidone clearly indicated, that LBH589 downregulates profibrotic gene expression while increasing apoptosis in IPF-fibroblasts, whereas pirfenidone-treatment maintained fibroblast-survival by effective suppression of pro-fibrotic phenotypes. Panobinostat thus revealed a favourable ‘inactivating’ effect against IPF-fibroblasts, and may have a significant benefit in treatment of IPF, as compared to pirfenidone. Although this study suggests that pirfenidone transforms an ‘abnormal’ IPF-fibroblast to an ‘altered lung fibroblast with approximately normal functions’, disease progression can only be decelerated by such strategy, presumably because IPF-fibroblasts are not ‘eliminated’ by pirfenidone. Since no curative treatment is yet available for IPF, the development of additional agents for this deadly disorder still represents a huge unmet need. We believe that pan-HDAC inhibition by LBH589 may present a novel therapeutic option for patients with IPF. The main observations and suggestions from this study are summarized in Fig 10.

## Conclusions

In conclusion, our study provides an overview and a direct head-to-head comparison of the effects of the neoplastic drug LBH589 and the IPF-drug pirfenidone on profibrogenic signaling in primary IPF-fibroblasts *in vitro*. Both drugs reduced to similar extent STAT3 activation, profibrotic gene expression and collagen-I-production. The strong HDAC inhibitor LBH589 clearly was more efficient than pirfenidone in inactivating IPF-fibroblasts, through induction of cell cycle arrest and apoptosis, an effect pirfenidone did not exert. Finally, we suggest that, beside other antifibrotic mechanisms, pirfenidone reduces profibrotic signaling also through weak epigenetic alterations in IPF-fibroblasts, but permits survival of ‘altered’ fibroblasts. The findings that pirfenidone-treatment favoured STAT3 inactivation as well as histone acetylation through down-regulation of HDAC enzymes, represent novel anti-fibrotic mechanisms of action for this drug.

## Supporting information

S1 Text Supplementary methods. RT-PCR and western blot.
(PDF)

S1 Table Primers used in semiquantitative RT-PCR (homo sapiens). (PDF)

S1 Fig Cellular consequences in primary IPF-fibroblasts in response to treatment with panobinostat or pirfenidone (supplemental data for Fig 3 of the manuscript). Primary IPF-fibroblasts (n = 5,6) were incubated for 24h with vehicle [Veh., 0.25% (v/v) DMSO], panobinostat (LBH589, 85 nM) or pirfenidone (Pirf., 2.7 mM). The effects of vehicle-, LBH589- and pirfenidone-treatment were analyzed by reverse transcription-polymerase chain reaction (RT-PCR) for indicated genes, and is depicted by representative agarose gels of RT-PCR products for *ACTA2*, *COL1A1*, *COL3A1*, *FN*, *CNN1*, *DES*, *P4HTM*, *CCND1*, *BIRC5*, *CIP1*, *P53*, *PUMA*, *DR5*, *ATF6*, *CHOP* and *CDKN2A*. *GAPDH* was used as reference gene. Results from two independent experiments are shown. -RT control = PCR of a RNA sample without reverse transcriptase.
(TIF)

S2 Fig Gene expression analysis for *BIRC5* and *HDAC9* in IPF- or normal fibroblasts in response to treatment with various HDAC inhibitors, sirtuin-1 activator resveratrol, and IPF-drug pirfenidone. (A) IPF-fibroblast cell line CCL-134 (n = 4) or (B) embryonic WI-38 fibroblasts (n = 4) were incubated for 24h with vehicle [Veh., 0.25% (v/v) DMSO, 0.1% (v/v) ethanol], panobinostat (LBH589, 85 nM, ‘LBH’), valproic acid (VPA, 1.5 mM), 4-phenyl-butyrate (4-PBA, 2 mM), resveratrol (Res., 90 μM) or pirfenidone (Pirf., 2.7 mM). Thereafter, cells were harvested and analyzed by qRT-PCR for *BIRC5* and *HDAC9*. *GAPDH* served as housekeeping gene. Data are presented as mean ± SEM of n = 4. *p<0.05 vs. vehicle; by Mann Whitney test.
(TIF)

S3 Fig Effects of LBH589- or pirfenidone treatment on histone deacetylase gene expression in primary IPF-fibroblasts (supplemental data for Fig 4 of the manuscript). Primary IPF-fibroblasts (n = 5,6) were incubated for 24h with vehicle [Veh., 0.25% (v/v) DMSO], panobinostat (LBH589, 85 nM) or pirfenidone (Pirf., 2.7 mM). The effects of vehicle-, LBH589- and pirfenidone-treatment were analyzed by semiquantitative reverse transcription-polymerase chain reaction (RT-PCR) for indicated HDAC genes, and is depicted by representative agarose gels of RT-PCR products for *HDAC1*, *HDAC2*, *HDAC3*, *HDAC4*, *HDAC5*, *HDAC6*, *HDAC7*, *HDAC8*, *HDAC9*, *HDAC10*, *HDAC11*, *SIRT1*, and *SIRT2*. Gene expression analysis for *HDAC9* was performed with n = 4/6 vehicle-, LBH589- and pirfenidone-treated IPF-fibroblasts. *GAPDH* was used as reference gene. Results from two independent experiments are shown. -RT control = PCR of a RNA sample without reverse transcriptase.
(TIF)

S4 Fig Localization of activated, phosphorylated STAT3 in idiopathic pulmonary fibrosis (IPF)- versus normal donor lungs. Representative immunohistochemistry for phosphorylated (p)-STAT3 (Y705), cytokeratin-5 (KRT5) and α-SMA in (A, C) IPF- and (B) normal donor lung tissue. (A, C) In IPF, the antibody for p-STAT3 revealed nuclear staining in myofibroblasts of fibroblast foci (indicated by α-SMA staining and dashed arrows in A and C) as well as in overlying abnormal bronchiolar basal cells [indicated by KRT5 expression in (A)]. (B) Normal donor lungs indicated no or minimal staining in the interstitium as well as alveolar epithelium.
(TIF)

S5 Fig Representative immunohistochemistry for KRT5, survivin, α-SMA, p-STAT3, and HDAC4 in serial sections of IPF-lung tissue. (A, B) Induction of p-STAT3 is observed in fibroblast foci (indicated by dashed arrows in A) and overlying abnormal bronchiolar epithelium (indicated by arrows and KRT5 expression in A), as well as in bronchioles of IPF-lungs (indicated by hashmark in B), and coincided with survivin and HDAC4 overexpression in these areas. Smooth muscle cells of IPF lungs (indicated by asterisk in B) also revealed nuclear p-STAT3 and survivin induction.
(TIF)

S6 Fig Protein expression analysis for α-SMA in IPF- or normal fibroblasts in response to treatment with various HDAC inhibitors, sirtuin-1 activator resveratrol, and IPF-drug pirfenidone. (A) IPF-fibroblast cell line CCL-134 (n = 4) or (B) embryonic WI-38 fibroblasts (n = 4) were incubated for 24h with vehicle [Veh., 0.25% (v/v) DMSO, 0.1% (v/v) ethanol], panobinostat (LBH589, 85 nM, ‘LBH’), valproic acid (VPA, 1.5 mM), 4-phenyl-butyrate (4-PBA, 2 mM), resveratrol (Res., 90 μM) or pirfenidone (Pirf., 2.7 mM). Thereafter, cells were harvested and analyzed by immunoblotting for α-SMA. GAPDH served as loading control. Data are presented as mean ± SEM of n = 4. *p<0.05 vs. vehicle; by Mann Whitney test.
(TIFF)

S7 Fig Effects of LBH589- or pirfenidone treatment on F-actin structures in primary IPF-fibroblasts. Primary IPF-fibroblasts (n = 3) were incubated for 24h with vehicle [Veh., 0.25% (v/v) DMSO], panobinostat (LBH589, 85 nM) or pirfenidone (Pirf., 2.7 mM), followed by fixation and staining with AlexaFluor 555-Phalloidin (red stain). Nuclei were counterstained with DAPI (blue stain). The cells were then analyzed by a fluorescence microscope. Vehicle-treated IPF-fibroblasts indicated beside linear F-actin structures stress fiber formation and extension of cells (left panel), which was impaired and abrogated in response to pirfenidone-treatment (right panel). In contrast to vehicle- (and pirfenidone-) treated cells, the panobinostat-treated IPF-fibroblasts revealed increased stress fiber formation in direction to a F-actin based cell expansion, resulting in a pronounced larger cell area and increased cell speading of single fibroblastic cells. Representative images for n = 3 IPF-fibroblast isolates are shown.
(TIF)

S8 Fig Uncropped western blots of figures Figs 1, 2, 5, 7 and 8. (PDF)
