# Supplementary material for: Comparison of the antifibrotic effects of the pan-histone deacetylase-inhibitor panobinostat versus the IPF-drug pirfenidone in fibroblasts from patients with idiopathic pulmonary fibrosis
Source: PLoS One. 2018 Nov 27;13(11):e0207915. doi: 10.1371/journal.pone.0207915 (PMC6258535; doi:10.1371/journal.pone.0207915)
Supplement: S1 Text — RT-PCR and western blot. (PDF) [file pone.0207915.s001.pdf]

Comparison of the antifibrotic effects of the pan-histone deacetylase-inhibitor panobinostat versus the IPF-drug pirfenidone in fibroblasts from patients with idiopathic pulmonary fibrosis

Martina Korfei[1,2]*; Daniel Stelmaszek[1,2]; BreAnne MacKenzie[1,2]; Sylwia Skwarna[1,2]; Shashipavan Chillappagari[1,2]; Anna C. Bach[1,2]; Clemens Ruppert[1,2,3]; Shigeki Saito[4]; Poornima Mahavadi[1,2]; Walter Klepetko[5,10]; Ludger Fink[2,3,6]; Werner Seeger[1,2,3,7]; Joseph A. Lasky[4]; Soni S. Pullamsetti[2,7]; Oliver H. Krämer[8]; Andreas Guenther[1,2,3,9,10].

***Corresponding Author**
Martina Korfei, Ph.D.
Department of Internal Medicine II, Justus-Liebig-University Giessen
Klinikstrasse 36, D-35392 Giessen, Germany
E-mail: Martina.Korfei@innere.med.uni-giessen.de
Tel.: +49 641 9942425
Fax: +49 641 9942429

Affiliation of co-authors:

[1]Department of Internal Medicine, Justus-Liebig-University Giessen, D-35392 Giessen, Germany.

[2]Universities of Giessen and Marburg Lung Center (UGMLC), Member of the German Center for Lung Research (DZL).

[3]Excellence Cluster Cardio-Pulmonary System (ECCPS), D-35392 Giessen, Germany.

[4]Department of Medicine, Section of Pulmonary Diseases, Critical Care and Environmental Medicine, Tulane University Health Sciences Center, New Orleans, LA 70112, USA.

[5]Department of Thoracic Surgery, Vienna General Hospital, A-1090 Vienna, Austria.

[6]Institute of Pathology and Cytology, D-35578 Wetzlar, Germany.

[7]Max-Planck-Institute for Heart and Lung Research, Department of Lung Development and Remodeling, D-61231 Bad Nauheim.

[8]Department of Toxicology, University Medical Center, D-55131 Mainz, Germany.

[9]Agaplesion Lung Clinic Waldhof Elgershausen, D-35753 Greifenstein, Germany.

[10]European IPF Network and European IPF Registry.

Supplementary Material and Methods

Western blot

Protein extracts from fibroblasts were prepared using a lysis buffer containing 50 mM Tris-HCl/pH 8.0, 5 mM EDTA, 150 mM NaCl, 1% (v/v) Triton-X-100, 0.5% (w/v) Na-deoxycholate and 1 mM PMSF. Protein extracts (15 µg per lane) were subjected for separation to 8-15% SDS-PAGE, followed by transfer onto PVDF membranes (Millipore) in a semi-dry blotting chamber according to the manufacturer´s protocol (Bio-Rad). Obtained immunoblots were then blocked by incubating at room temperature (RT) for 1h in blocking buffer [1 × tris-buffered saline (TBS; 50 mM tris-HCl, pH 7.5, 50 mM NaCl) containing 5% (w/v) nonfat dried milk and 0.1% (v/v) tween 20], followed by immunostaining for the proteins of interest. In the following, the primary antibodies used for immunoblotting are listed, including the sources and dilutions: mouse monoclonal for human HDAC1 (1:200, Santa Cruz, sc-81598), rabbit monoclonal for human HDAC2 (1:2000, Abcam, ab32117), rabbit polyclonal for human HDAC6 (1:300, abcam, ab133493), rabbit polyclonal for human HDAC9 (1:300, Santa Cruz, sc-28732), rabbit polyclonal for human histone H3 [acetyl K27] (1:15000 [or 1:2000 in case of balanced acetylation], Abcam, ab4729), rabbit polyclonal for human histone H3 (1:10000, Abcam, ab1791), mouse monoclonal for acetylated tubulin [from the outer arm of *Strongylocentrotus purpuratus* (sea urchin)] (1:60000 [or 1:2000 in case of balanced acetylation], Sigma, T7451), mouse monoclonal for human p21 (1:250, Abcam, ab16767), mouse monoclonal for human cyclin D1 (1:250, Abcam, ab10540), rabbit polyclonal for human survivin (1:750, Abcam, ab24479), mouse monoclonal for human phospho-STAT3 [Y705] (1:300, Cell Signaling Technology, #4113S), mouse monoclonal for human STAT3 (1:300, Cell Signaling Technology, #9139S), rabbit polyclonal for human phospho-histone H3

(1:500, Abcam, ab5176), rabbit polyclonal for human Bcl-XL (1:1000, Abcam, ab32370), rabbit polyclonal for human CHOP/GADD153 (1:500, Santa Cruz, sc-793), rabbit polyclonal for human DR5 (1:500, Abcam, ab47179), goat polyclonal for mouse AIF, mitochondrial (1:1000, Santa Cruz, sc-9416), mouse monoclonal for human α-SMA (1:15000, Abcam, ab119952), rabbit monoclonal for human tropomyosin 1 (1:500, Abcam, ab133292), rabbit polyclonal for human collagen α1-type I [COL1A1] (1:3000, Rockland, #600-401-103), mouse monoclonal for human p53 (1:250, Santa Cruz, sc-263), rabbit polyclonal for human caspase-3 (1:500, Cell Signaling Technology, #9662), rabbit monoclonal for human cleaved caspase-3 [Asp175] (1:300, Cell Signaling Technology, #9664), rabbit monoclonal for human cleaved PARP1-p25 (1:500, Abcam, ab32064), and rabbit monoclonal for human p16INK4a (1:300, Abcam, ab108349). The blots were then washed four times in 1 × TBS containing 0.1 % (w/v) tween 20, and incubated with respective horseradish peroxidase (HRP)-conjugated secondary antibodies (DakoCytomation, Hamburg, Germany; rabbit anti-mouse-IgG, rabbit anti-goat-IgG, or swine anti-rabbit IgG, all diluted 1:2000 in blocking buffer) for 2 hours at room temparature. After four washes, blot membranes were developed with the Immobilon Western Chemiluminescent HRP substrate (Millipore), and emitted signals were detected with a chemiluminescence imager (Intas ChemoStar, Germany). Thereafter, blots were stripped using ″stripping buffer″ [2% (w/v) SDS and 50mM dithiothreitol in 1 × TBS] under gentle shaking at 60°C for 45 min, followed by re-probing the blots with antibodies against the loading control GAPDH (goat polyclonal or mouse monoclonal for human GAPDH, Santa Cruz, sc-20358 and sc-47724, respectively, both diluted 1:2000) or β-actin (mouse monoclonal for human β-actin, Abcam, ab8226, diluted 1:3000). For quantification, band intensities were quantified by densitometry using

ImageJ software (Version 1.46r, NIH). The band densities were normalized to loading controls.

Analysis of acetylation status of histone H3-K27 and α-tubulin in vehicle-, LBH589- and pirfenidone-treated IPF-fibroblasts

Due to highly increased acetylation of this core histone in LBH589-treated IPF-fibroblasts in comparison to vehicle and pirfenidone, we had to develop the respective western blot in a very short exposure time (20 sec.) to show an adequate staining for H3K27Ac in LBH589-treatments, despite use of the anti-H3K27Ac antibody (Abcam, ab4729) in a dilution of 1:15000, and thus detection of H3K27Ac in the other treatments could not be visualized within a 20 sec. exposure time (Fig 1A, main manuscript). We therefore repeated the immunoblot by using only fibroblast-lysates of vehicle- and pirfendone-treatment, and omitting the LBH589-lysates. Because we expected only low amounts of H3K27Ac in both conditions, we used the respective antibody in a dilution of 1:2000 and developed the blot after a longer exposure of the luminescence signals (2 min). As shown in Fig 1B (main manuscript), pirfenidone led also to a significant increase of H3K27-acetylation in IPF-fibroblasts, when compared to vehicle.

In addition, LBH589 also resulted in a very strong increase of tubulin-acetylation in IPF-fibroblasts, as compared to vehicle and pirfenidone, and which was (similar to H3K27Ac) already detected after a short exposure time (20 sec.) by using paradoxically a very low concentration of the anti-acetylated α-tubulin antibody (1:60000) (Fig 1C, main manuscript). Again, a clear evaluation of tubulin-acetylation status in vehicle- versus pirfenidone-treated IPF-fibroblasts could not be made, and an additional immunoblot of only vehicle- and pirfenidone-treated IPF-fibroblasts was performed. As shown in Fig 1D (main manuscript), the use of the anti-acetylated α-tubulin antibody in the dilution 1:2000 and a longer exposure time of 2 min revealed

basal levels of acetylated α-tubulin in vehicle-, but significantly diminished tubulin-acetylation in pirfenidone-treated IPF-fibroblasts.

Reverse Transcription-Polymerase Chain Reaction (RT-PCR)

Total cellular RNA was prepared from treated IPF-fibroblasts using the RNeasy® Plus Mini-Kit (Qiagen) and 600 µL RLT Plus-lysis buffer according to the protocol of the manufacturer. The purity and quantity of the isolated RNA was determined by spectrophotometry at 260/280 nm using NanoDrop 2000c photometer (PeqLab).

Reverse transcription (RT) and PCR were performed sequentially in two separate steps. Complementary DNA (cDNA, 2 µg) was first synthesized by reverse transcription (RT) using 2 µg total RNA, and with use of oligo-dT-primers and the Omniscript-RT-Kit (Qiagen). An aliquot of the finished RT reaction/cDNA (100 ng) was then used for PCR amplification employing gene-specific primers for transcripts. The complete list of primers used is given in S1 Table.

Each 40-µL RT reaction contained (final concentration): 2 µg total RNA, 1 µM oligo-dT-primers, 10 units RNAse inhibitor (both Applied Biosystems), 500 µM of each dNTP and 4 units Omniscript Reverse Transcriptase (both Qiagen). The RT reactions were incubated for 65 min at 37°C, directly followed by PCR using gene-specific primers. Each 20-µL PCR reaction contained (final concentration): 2 µL finished RT reaction (= 100 ng template cDNA), 0.2 µM each forward and reverse primer (metabion, Martinsried, Germany), 200 µM of each dNTP (Thermo Scientific) and 0.4 µL Phire-Hot-Start-II-DNA-Polymerase (Thermo Scientific). For amplification of all described *c*DNAs/genes, following cycling protocol according to the manufacturer´s protocol was performed: ″Hot-Start″ (initial activation step: 98°C for 30 sec.) followed by 3-step-cycling (20-35 cycles of amplification): denaturation: 98°C for 5 sec., annealing: 59-65°C for 5 sec., extension: 72°C for 15 sec. (and 60 sec. in the final

extension). The thermal cycler used was from Bio-Rad (model: PTC-1148). In addition, the size of the amplified PCR product for each gene/*c*DNA, and the number of cycles for amplification of each *c*DNA, are given in S1 Table. As control experiment, PCR reactions of RNA samples without reverse transcriptase were performed (to exclude amplification of genomic DNA contaminations).

Equal aliquots of the PCR products were electrophoresed through a 2% (w/v) agarose gel containing ethidium bromide in 1× tris-acetate-EDTA (TAE) buffer, and documented by scanning using an UV imager (Gel-Doc XR$^+$, Bio-Rad). Thereafter, band intensities of PCR products were quantified using Image Lab-Software (version 5.2.1, Bio-Rad), and mRNA expression of genes of interest were normalized to the expression of *GAPDH*.
