# Supplementary material for: Comparison of the antifibrotic effects of the pan-histone deacetylase-inhibitor panobinostat versus the IPF-drug pirfenidone in fibroblasts from patients with idiopathic pulmonary fibrosis
Source: PLoS One. 2018 Nov 27;13(11):e0207915. doi: 10.1371/journal.pone.0207915 (PMC6258535; doi:10.1371/journal.pone.0207915)
Supplement: S1 Table — (PDF) [file pone.0207915.s002.pdf]

S1 Table: Primers used in semiquantitative RT-PCR (homo sapiens)

Gene/Name of primer	Primer-Sequence	Annealing Temp.(T_A) *	Size of PCR product	No. of Cycles
HDAC1 forward	5´- GCC GCA AGA ACT CTT CCA AC - 3´	63°C	140 bp	28
HDAC1 reverse	5´- CTT GAC CCC TTT GGC TTC TG - 3´			
HDAC2 forward	5´- CAG GAG ACT TGA GGG ATA TTG G - 3´	65°C	320 bp	25
HDAC2 reverse	5´- ATG TGT CCA ACA TCG AGC AAC - 3´			
HDAC3 forward	5´- CCG GTT ATC AAC CAG GTA GTG - 3´	64°C	321 bp	25
HDAC3 reverse	5´- GGT GCT GAC ATC TGG ATG AAG - 3´			
HDAC4 forward	5´- CTT CTG CAG CAG AGG TTG AG - 3´	63°C	267 bp	28
HDAC4 reverse	5´- TCT GAA GGC CGC CAA GTA C - 3´			
HDAC5 forward	5´- TCA ACC GGC AGA AGC TAG AC - 3´	63°C	319 bp	27
HDAC5 reverse	5´- TTG CCC ACG TTC AAC TTC TG - 3´			
HDAC6 forward	5´- CCC AGC ACA GTC TTA TGG - 3´	63°C	320 bp	29
HDAC6 reverse	5´- AGG AAA GCA GCA ATG TAG - 3´			
HDAC7 forward	5´- CTC AAA CTG GAC AAC GGG AAG - 3´	63°C	270 bp	27
HDAC7 reverse	5´- GGC CAC TGA GTT GAA GAA GC - 3´			
HDAC8 forward	5´- GTC CCG AGT ATG TCA GTA TGT G - 3´	65°C	222 bp	27
HDAC8 reverse	5´- CTA TGG AGT CCG GAT GAT CAT C - 3´			
HDAC9 forward	5´- GAA AGG GCA GTG GCA AGT AC - 3´	63°C	222 bp	29
HDAC9 reverse	5´- ATC TTG TGC TCC TGG TAA TGT G - 3´			
HDAC10 forward	5´- GCA GGT GAA CAG TGG TAT AGC - 3´	63°C	220 bp	28
HDAC10 reverse	5´- CGT GGA GAC ATG GAA CAT GG - 3´			
HDAC11 forward	5´- AGG GCT ACC ATC ATT GAT CTT G - 3´	63°C	222 bp	28
HDAC11 reverse	5´- CTG GAG GGA TTT CTT GAT GTT C - 3´			
SIRT1 forward	5´- CAA GCT CTA GTG ACT GGA CTC - 3´	64°C	321 bp	30
SIRT1 reverse	5´- CAT CCC TTG ACC TGA AGT CAG- 3´			
SIRT2 forward	5´- CGG TAC ATG CAG AGC GAA C - 3´	60°C	221 bp	27
SIRT2 reverse	5´- TGC CCA GGA TAG AGT TCC TTG - 3´			
GAPDH forward	5´- ACC CAG AAG ACT GTG GAT GG - 3´	59°C	320 bp	24
GAPDH reverse	5´- GTG TCG CTG TTG AAG TCA GAG - 3´			
DR5 forward	5´- AAG ACG GTA GAG ATT GCA - 3´	62°C	*401 bp<br>*299 bp	28
DR5 reverse	5´- CTA CGG CTG CAA CTG TGA C - 3´			
P53 forward	5´- CCT CAG CAT CTT ATC CGA GTG - 3´	64°C	223 bp	25
P53 reverse	5´- GTA GAT TAC CAC TGG AGT CTT CC – 3´			
CIP1 forward	5´- GAT GGA ACT TCG ACT TTG TCA C – 3´	61°C	220 bp	25
CIP1 reverse	5´- GGC ACA AGG GTA CAA GAC AG – 3´			
PUMA forward	5´- ATG GCG GAC GAC CTC AAC – 3´	62°C	119 bp	26
PUMA reverse	5´- CTG GGT AAG GGC AGG AGT C – 3´			
ACTA2 forward	5´- GAG ATC TCA CTG ACT ACC TCA TG – 3´	60°C	269 bp	26
ACTA2 reverse	5´- AGC AGA CTC CAT CCC GAT G - 3´			
COL1A1 forward	5´- TGC CAC TCT GAC TGG AAG AG – 3´	60°C	320 bp	20
COL1A1 reverse	5´- TTG CAG TGG TAG GTG ATG TTC- 3´			
COL3A1 forward	5´- TTA CAA GGC TTA CCT GGT ACA G - 3´	59°C	269 bp	25
COL3A1 reverse	5´- CCA GGC ATT CCT TGC AGA C - 3´			
FN forward	5´- CAT ACC ACG TAG GAG AAC AGT G - 3´	62°C	221 bp	20
FN reverse	5´- AGG CAT GAA GCA CTC AAT TGG - 3´			
CNN1 forward	5´- GAG TGA AGT ACG CAG AGA AGC - 3´	59°C	269 bp	26
CNN1 reverse	5´- CTC GAA GAT CTG CCG CTT G - 3´			

S1 Table continued: Primers used in semiquantitative RT-PCR (homo sapiens)

Gene/Name of primer	Primer-Sequence	Annealing Temp.(T_A) *	Size of PCR product	No. of Cycles
P4HTM forward	5´- TGG ATT ACC TGC CAG AGA GAC - 3´	64°C	272 bp	26
P4HTM reverse	5´- AGT CTC AGG GTC ATG TAC TGT AG - 3´			
DES forward	5´- GAA TCT CTC AAC GAG GAG ATC G-3´	60°C	270 bp	28
DES reverse	5´- GGT CTG ACA CCT TCG ACT TG - 3´			
CCND1 forward	5´- CGA GAA GCT GTG CAT CTA CAC - 3´	62°C	221 bp	26
CCND1 reverse	5´- ACT TCA CAT CTG TGG CAC AAG - 3´			
BIRC5 forward	5´- AAA GCA TTC GTC CGG TTG C - 3´	57°C	161 bp	25
BIRC5 reverse	5´- GCA CTT TCT TCG CAG TTT CC - 3´			
CDKN2A forward	5´- ACC AGA GGC AGT AAC CAT GC - 3´ #	60°C	122 bp	28
CDKN2A reverse	5´- CCT GTA GGA CCT TCG GTG AC - 3´ #			
ATF6 forward	5´- ACT CAG GGA GTG AGC TAC AAG - 3´	64°C	270 bp	26
ATF6 reverse	5´- GGA GGA TCC TGG TGT CCA TC - 3´			
CHOP forward	5´- ACT CTC CAG ATT CCA GTC AGA G - 3´	61°C	220 bp	25
CHOP reverse	5´- GCC TCT ACT TCC CTG GTC AG - 3´			

*It has to be noted that the annealing temperature (T_A) for listed primers is only valid for Phire-Hot-Start-II-DNA-Polymerase. The annealing rules for Phire II are different from many common DNA polymerases (such as *Taq* DNA polymerases). As a basic rule, for primers > 20 nt, the T_A is T_m +3°C of the lower T_m primer. For primers ≤ 20 nt, the T_A has to be equal to the T_m of the lower T_m primer.
T_m = melting temperature.
*two PCR products due to alternative splicing

With the exception of *CDKN2A* (p16), primers were designed using "GeneFisher/GeneFisher2"-Software - interactive PCR primer design. (http://bibiserv.techfak.unibielefeld.de/genefisher2/).
GeneFisher - software support for the detection of postulated genes. *Robert Giegerich, Folker Meyer and Chris Schleiermacher.* Proc Int Conf Intell Syst Mol Biol. 1996;4:68-77.
In the majority, intron-spanning primers were chosen.

Lehmann M, Korfei M, Mutze K, Klee S, Skronska-Wasek W, Alsafadi HN, Ota C, Costa R, Schiller HB, Lindner M, Wagner DE, Gunther A, Konigshoff M. Senolytic drugs target alveolar epithelial cell function and attenuate experimental lung fibrosis ex vivo. Eur Respir J 2017;50.
