# Supplementary figures and images for: Comparison of the antifibrotic effects of the pan-histone deacetylase-inhibitor panobinostat versus the IPF-drug pirfenidone in fibroblasts from patients with idiopathic pulmonary fibrosis
Source: PLoS One. 2018 Nov 27;13(11):e0207915. doi: 10.1371/journal.pone.0207915 (PMC6258535; doi:10.1371/journal.pone.0207915)

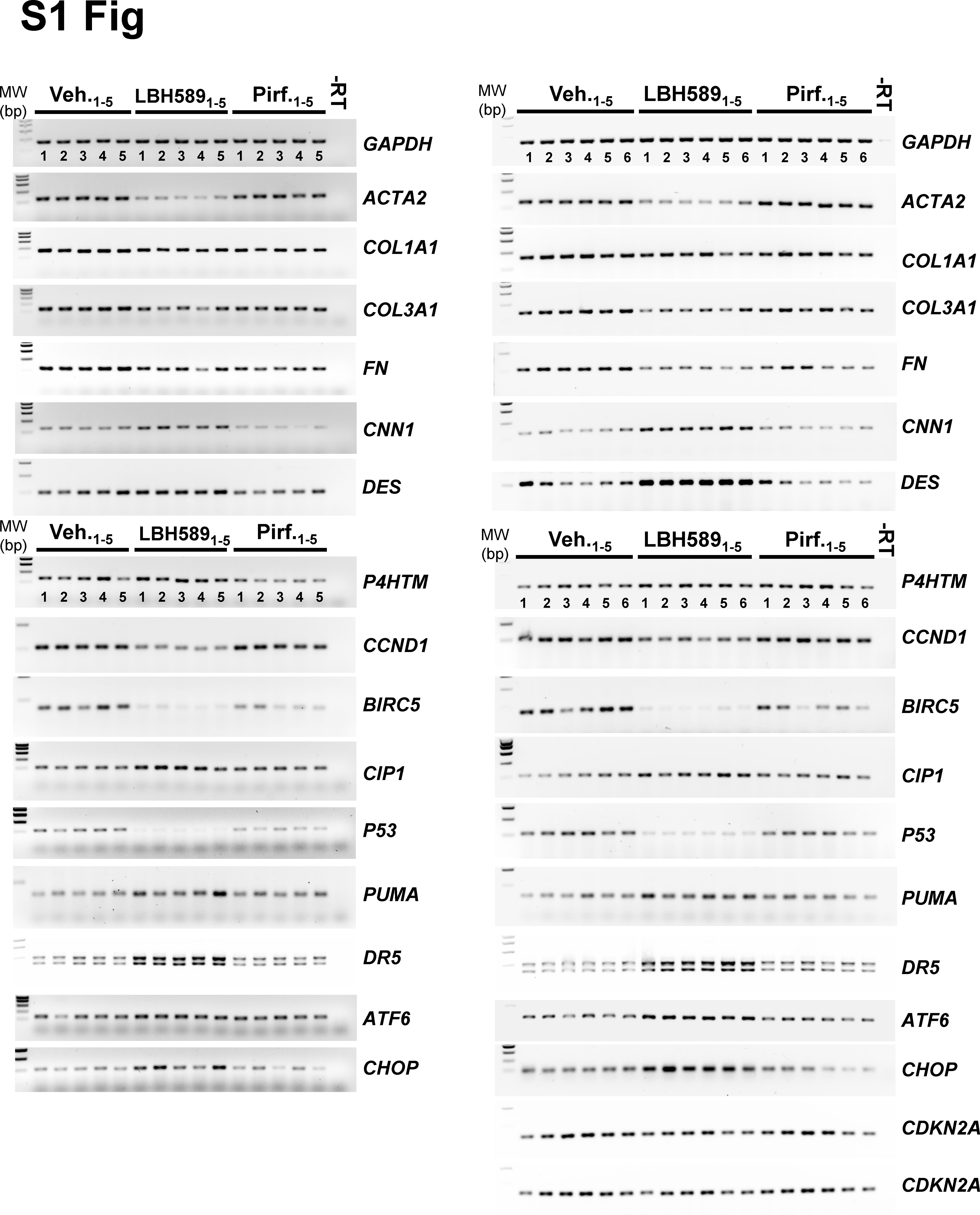

Supplement: S1 Fig — Primary IPF-fibroblasts (n = 5,6) were incubated for 24h with vehicle [Veh., 0.25% (v/v) DMSO], panobinostat (LBH589, 85 nM) or pirfenidone (Pirf., 2.7 mM). The effects of vehicle-, LBH589- and pirfenidone-treatment were analyzed by reverse transcription-polymerase chain reaction (RT-PCR) for indicated genes, and is depicted by representative agarose gels of RT-PCR products for ACTA2, COL1A1, COL3A1, FN, CNN1, DES, P4HTM, CCND1, BIRC5, CIP1, P53, PUMA, DR5, ATF6, CHOP and CDKN2A. GAPDH was used as reference gene. Results from two independent experiments are shown. -RT control = PCR of a RNA sample without reverse transcriptase. (TIF) [file pone.0207915.s003.tif]

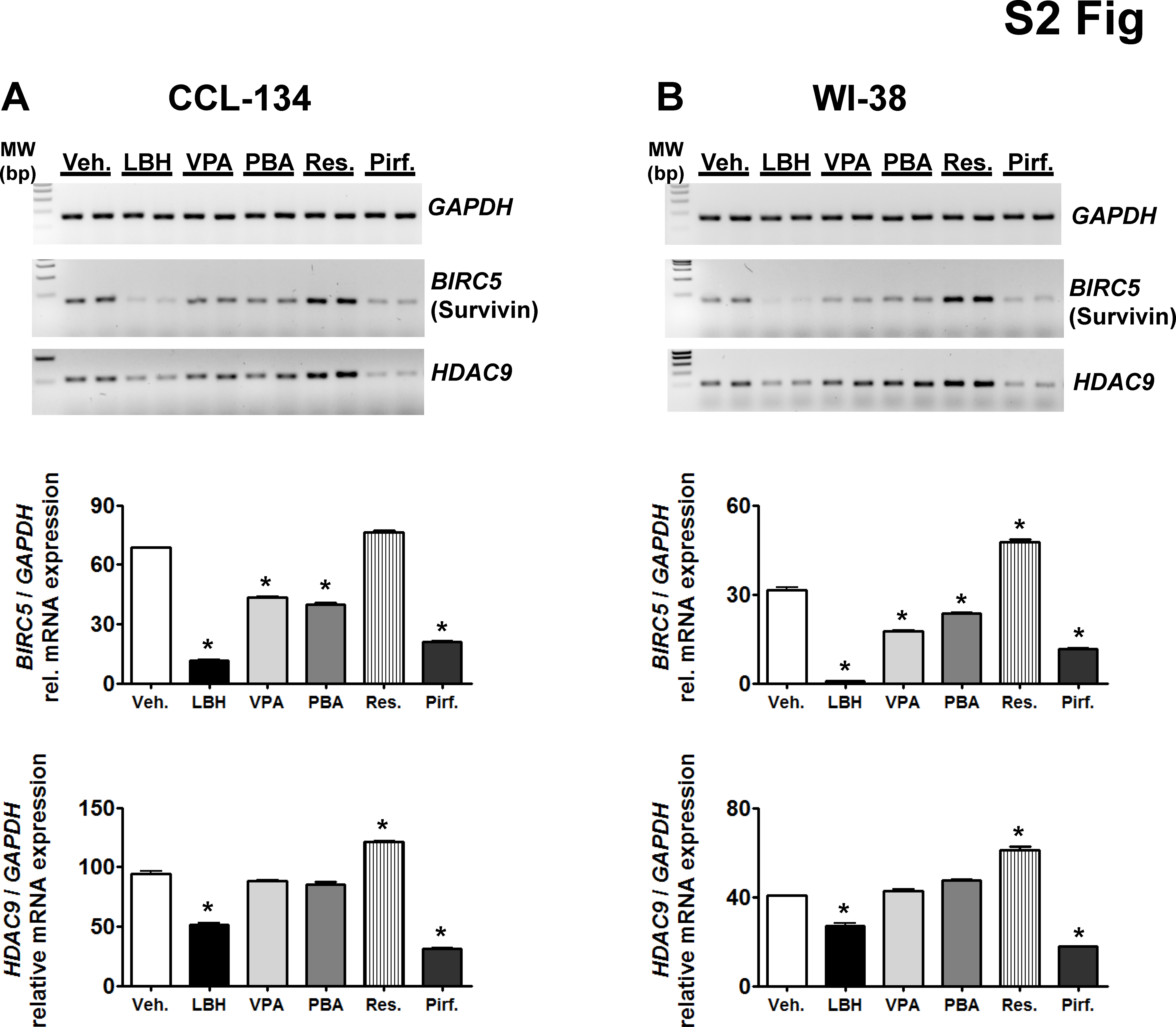

Supplement: S2 Fig — (A) IPF-fibroblast cell line CCL-134 (n = 4) or (B) embryonic WI-38 fibroblasts (n = 4) were incubated for 24h with vehicle [Veh., 0.25% (v/v) DMSO, 0.1% (v/v) ethanol], panobinostat (LBH589, 85 nM, ‘LBH’), valproic acid (VPA, 1.5 mM), 4-phenyl-butyrate (4-PBA, 2 mM), resveratrol (Res., 90 μM) or pirfenidone (Pirf., 2.7 mM). Thereafter, cells were harvested and analyzed by qRT-PCR for BIRC5 and HDAC9. GAPDH served as housekeeping gene. Data are presented as mean ± SEM of n = 4. *p<0.05 vs. vehicle; by Mann Whitney test. (TIF) [file pone.0207915.s004.tif]

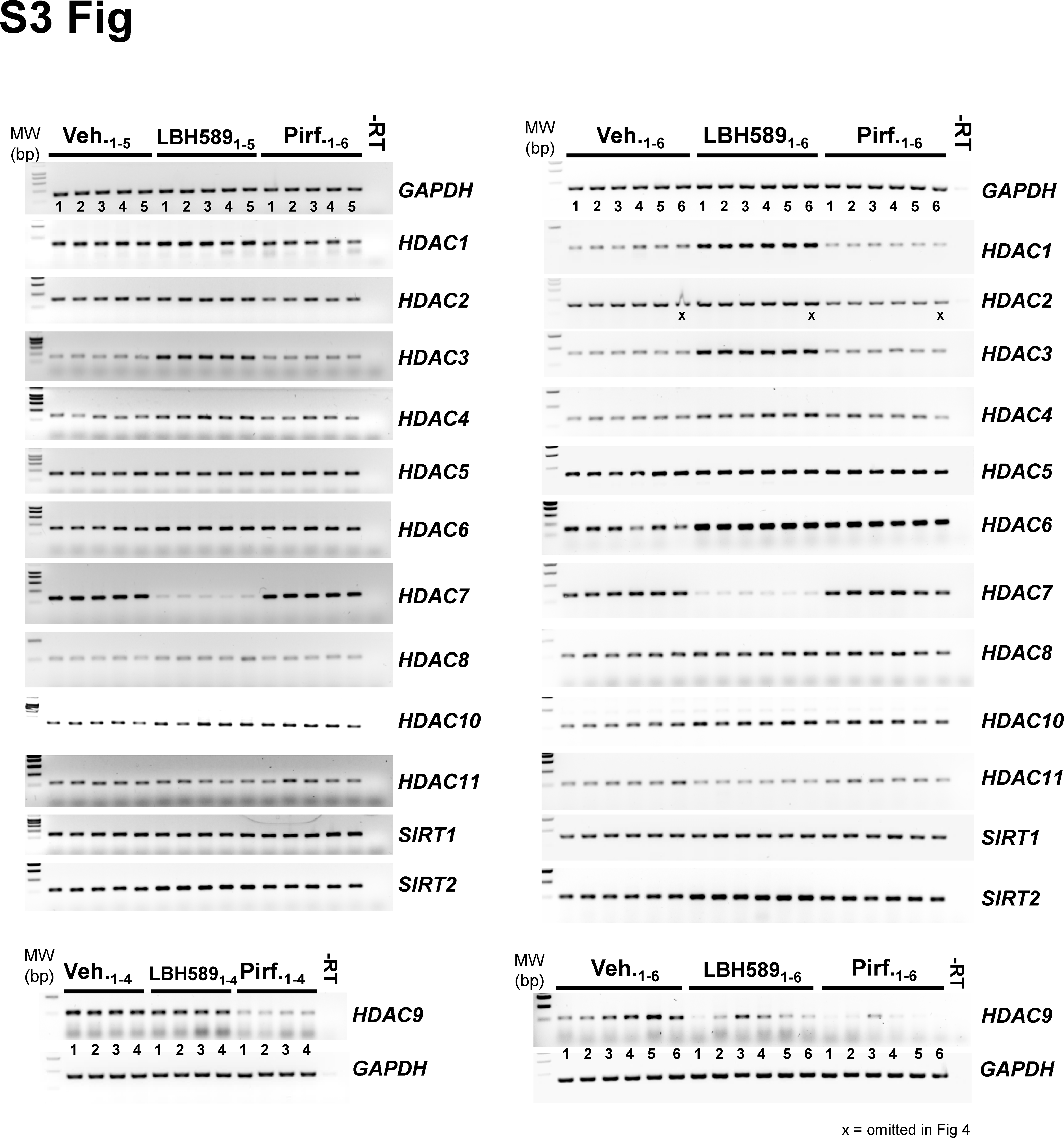

Supplement: S3 Fig — Primary IPF-fibroblasts (n = 5,6) were incubated for 24h with vehicle [Veh., 0.25% (v/v) DMSO], panobinostat (LBH589, 85 nM) or pirfenidone (Pirf., 2.7 mM). The effects of vehicle-, LBH589- and pirfenidone-treatment were analyzed by semiquantitative reverse transcription-polymerase chain reaction (RT-PCR) for indicated HDAC genes, and is depicted by representative agarose gels of RT-PCR products for HDAC1, HDAC2, HDAC3, HDAC4, HDAC5, HDAC6, HDAC7, HDAC8, HDAC9, HDAC10, HDAC11, SIRT1, and SIRT2. Gene expression analysis for HDAC9 was performed with n = 4/6 vehicle-, LBH589- and pirfenidone-treated IPF-fibroblasts. GAPDH was used as reference gene. Results from two independent experiments are shown. -RT control = PCR of a RNA sample without reverse transcriptase. (TIF) [file pone.0207915.s005.tif]

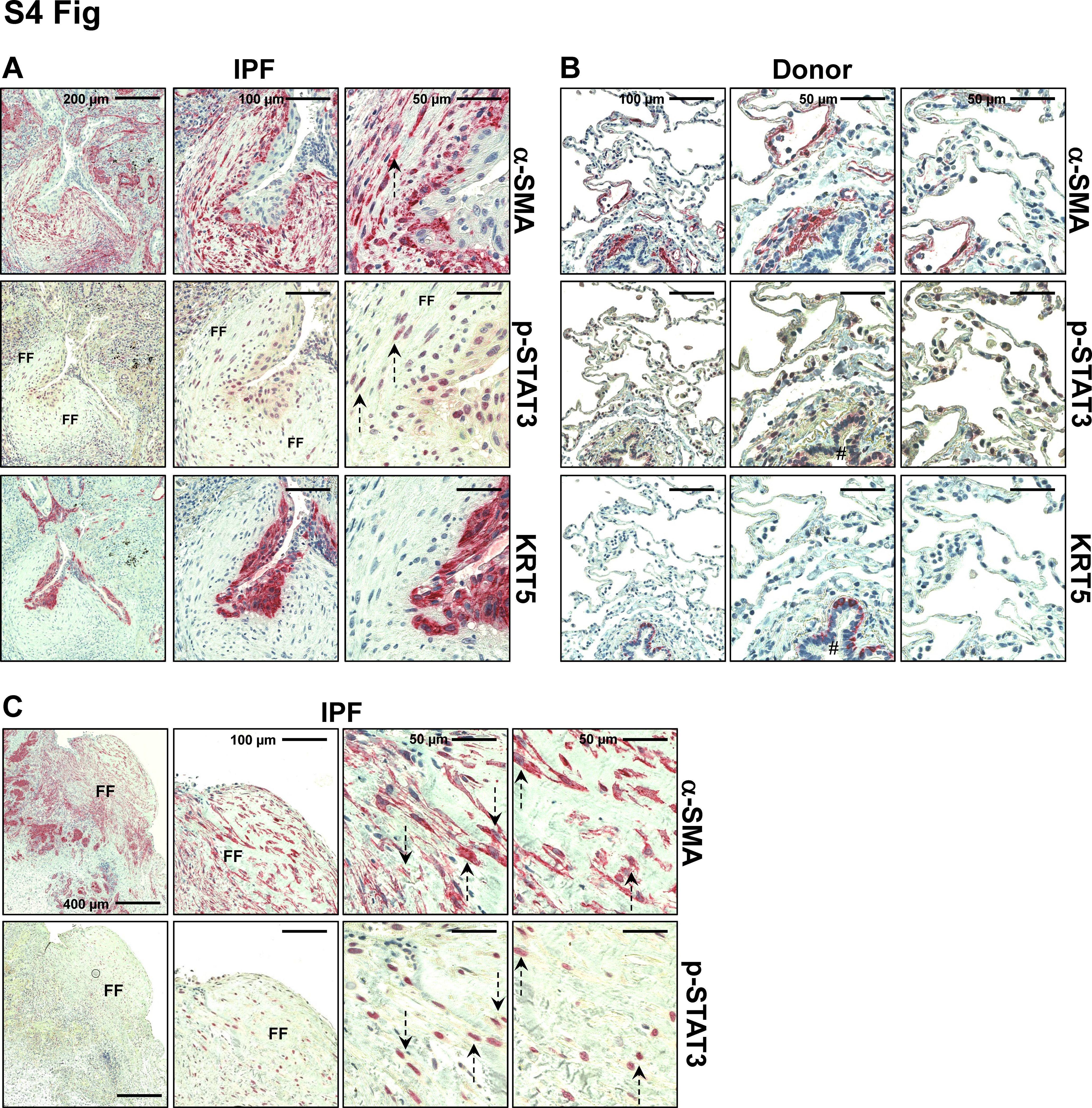

Supplement: S4 Fig — Representative immunohistochemistry for phosphorylated (p)-STAT3 (Y705), cytokeratin-5 (KRT5) and α-SMA in (A, C) IPF- and (B) normal donor lung tissue. (A, C) In IPF, the antibody for p-STAT3 revealed nuclear staining in myofibroblasts of fibroblast foci (indicated by α-SMA staining and dashed arrows in A and C) as well as in overlying abnormal bronchiolar basal cells [indicated by KRT5 expression in (A)]. (B) Normal donor lungs indicated no or minimal staining in the interstitium as well as alveolar epithelium. (TIF) [file pone.0207915.s006.tif]

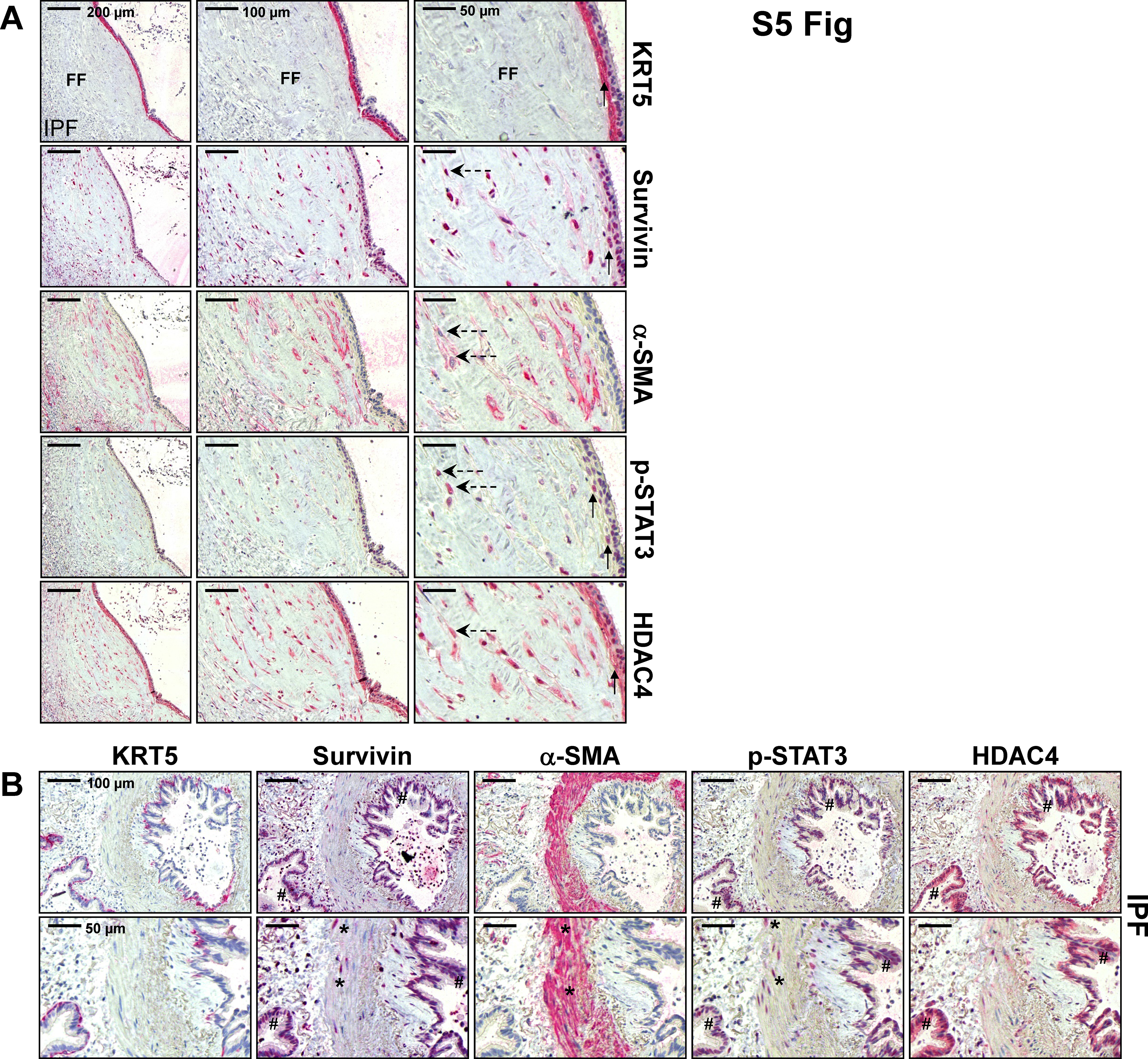

Supplement: S5 Fig — (A, B) Induction of p-STAT3 is observed in fibroblast foci (indicated by dashed arrows in A) and overlying abnormal bronchiolar epithelium (indicated by arrows and KRT5 expression in A), as well as in bronchioles of IPF-lungs (indicated by hashmark in B), and coincided with survivin and HDAC4 overexpression in these areas. Smooth muscle cells of IPF lungs (indicated by asterisk in B) also revealed nuclear p-STAT3 and survivin induction. (TIF) [file pone.0207915.s007.tif]

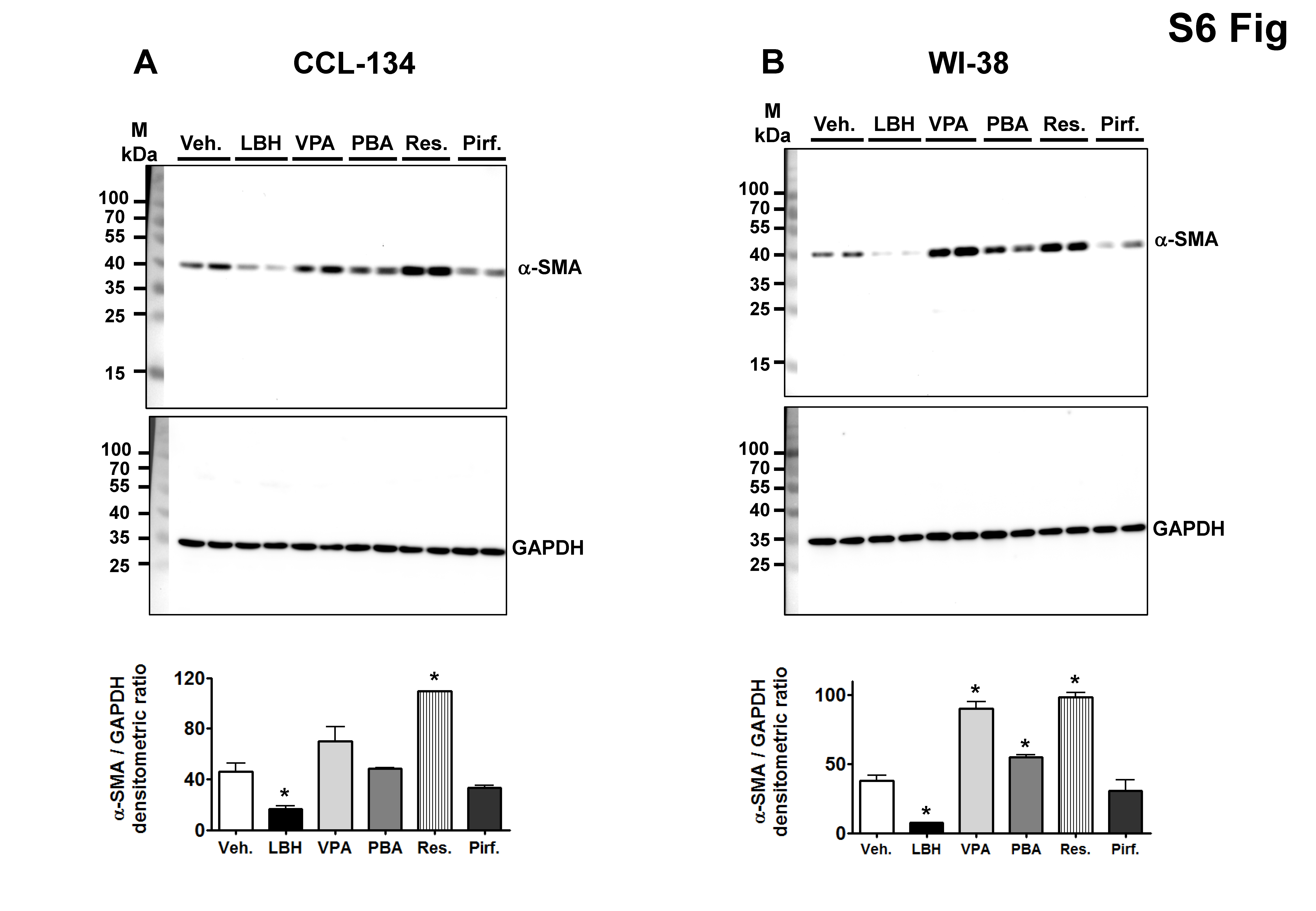

Supplement: S6 Fig — (A) IPF-fibroblast cell line CCL-134 (n = 4) or (B) embryonic WI-38 fibroblasts (n = 4) were incubated for 24h with vehicle [Veh., 0.25% (v/v) DMSO, 0.1% (v/v) ethanol], panobinostat (LBH589, 85 nM, ‘LBH’), valproic acid (VPA, 1.5 mM), 4-phenyl-butyrate (4-PBA, 2 mM), resveratrol (Res., 90 μM) or pirfenidone (Pirf., 2.7 mM). Thereafter, cells were harvested and analyzed by immunoblotting for α-SMA. GAPDH served as loading control. Data are presented as mean ± SEM of n = 4. *p<0.05 vs. vehicle; by Mann Whitney test. (TIFF) [file pone.0207915.s008.tiff]

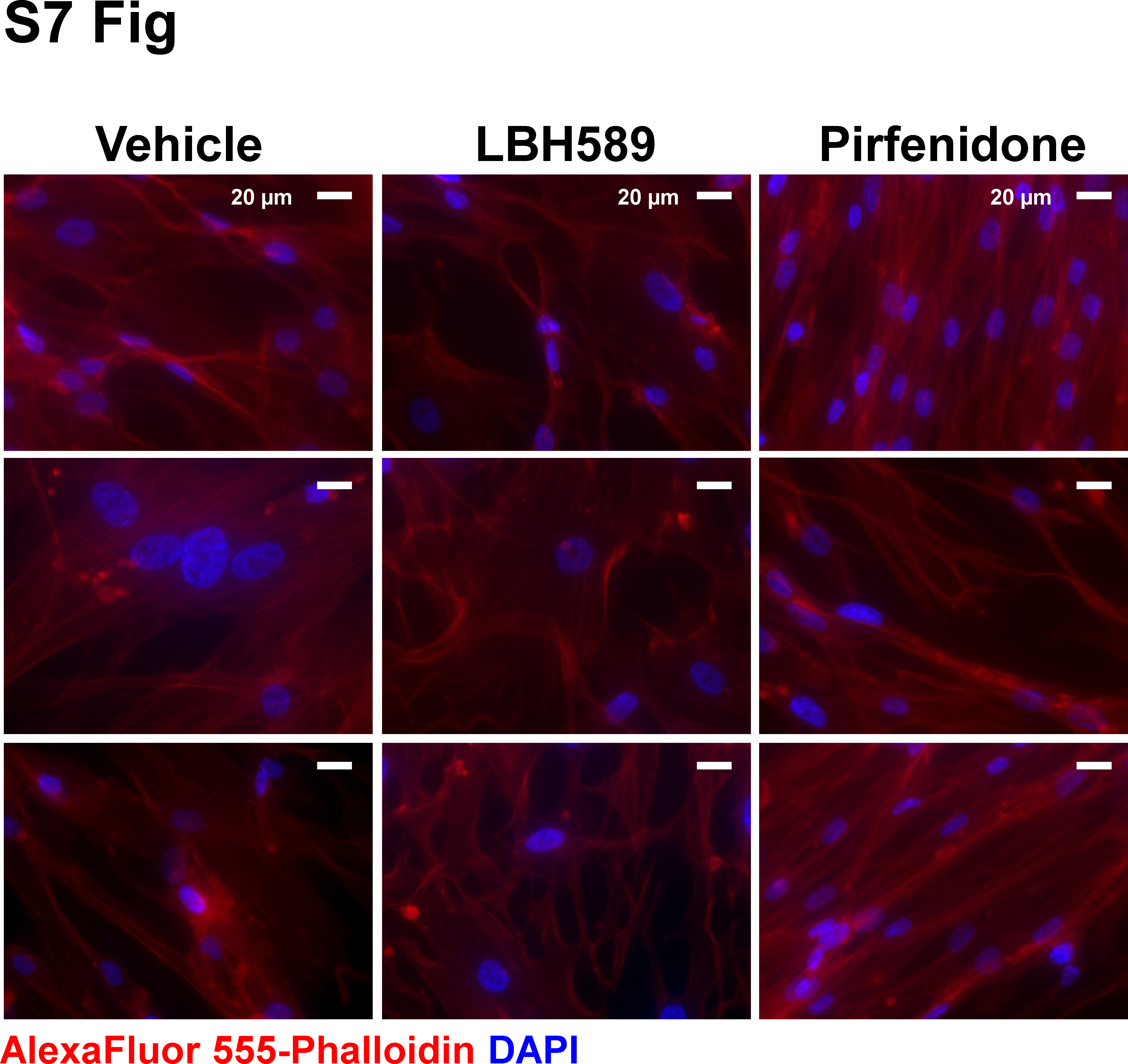

Supplement: S7 Fig — Primary IPF-fibroblasts (n = 3) were incubated for 24h with vehicle [Veh., 0.25% (v/v) DMSO], panobinostat (LBH589, 85 nM) or pirfenidone (Pirf., 2.7 mM), followed by fixation and staining with AlexaFluor 555-Phalloidin (red stain). Nuclei were counterstained with DAPI (blue stain). The cells were then analyzed by a fluorescence microscope. Vehicle-treated IPF-fibroblasts indicated beside linear F-actin structures stress fiber formation and extension of cells (left panel), which was impaired and abrogated in response to pirfenidone-treatment (right panel). In contrast to vehicle- (and pirfenidone-) treated cells, the panobinostat-treated IPF-fibroblasts revealed increased stress fiber formation in direction to a F-actin based cell expansion, resulting in a pronounced larger cell area and increased cell speading of single fibroblastic cells. Representative images for n = 3 IPF-fibroblast isolates are shown. (TIF) [file pone.0207915.s009.tif]
