# Supplementary material for: Comparison of the antifibrotic effects of the pan-histone deacetylase-inhibitor panobinostat versus the IPF-drug pirfenidone in fibroblasts from patients with idiopathic pulmonary fibrosis
Source: PLoS One. 2018 Nov 27;13(11):e0207915. doi: 10.1371/journal.pone.0207915 (PMC6258535; doi:10.1371/journal.pone.0207915)
Supplement: S8 Fig — (PDF) [file pone.0207915.s010.pdf]

S8 Fig Uncropped western blots

Fig 1

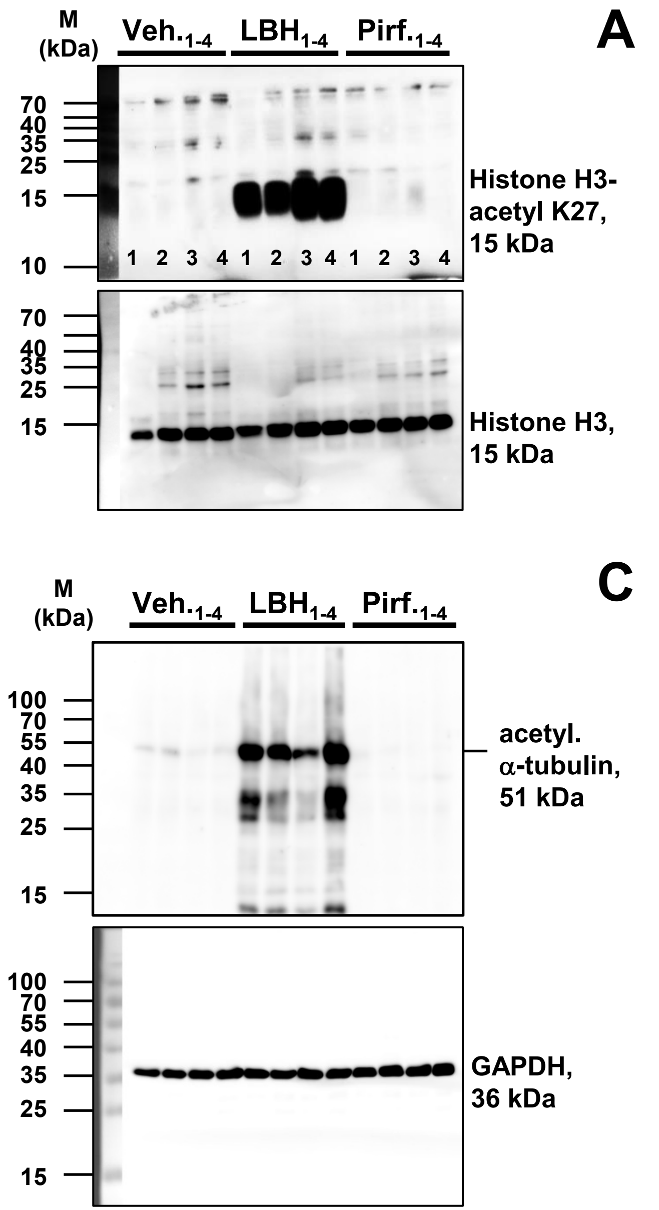

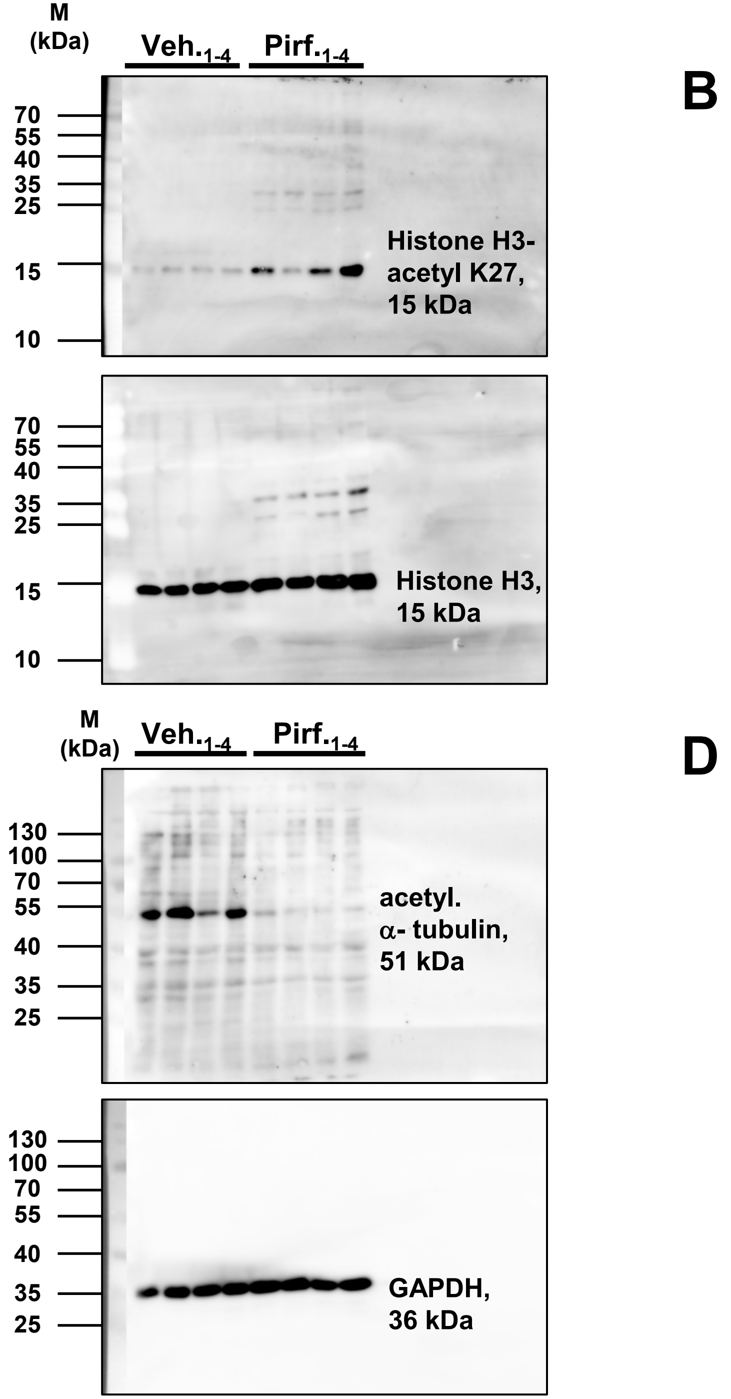

Fig 2

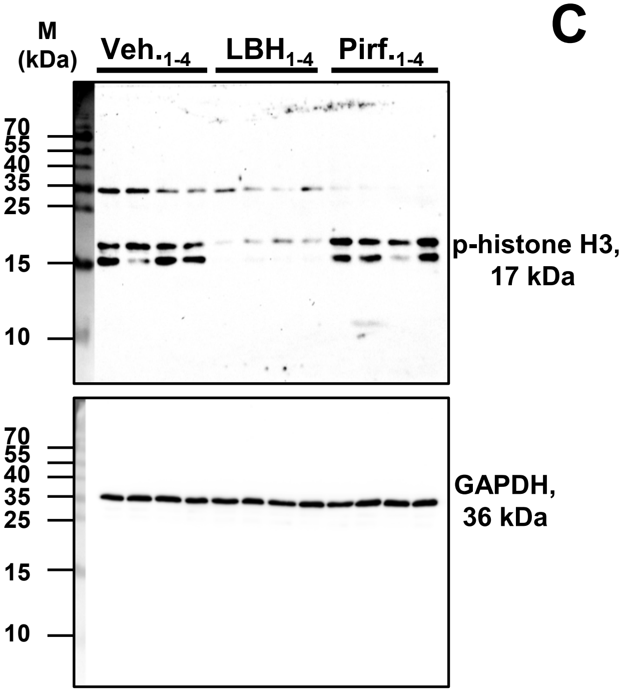

C
M
(kDa)
Veh.1-4
LBH1-4
Pirf.1-4
70
55
40
35
25
15
10
p-histone H3,
17 kDa
70
55
40
35
25
15
10
GAPDH,
36 kDa

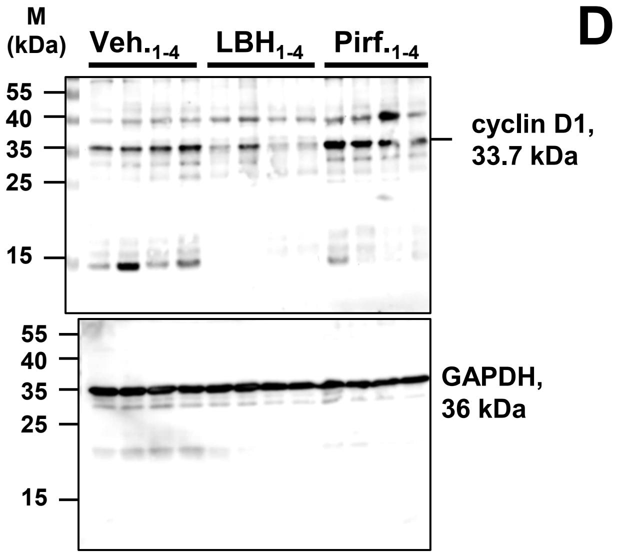

D
M
(kDa)
Veh.1-4
LBH1-4
Pirf.1-4
55
40
35
25
15
cyclin D1,
33.7 kDa
55
40
35
25
15
GAPDH,
36 kDa

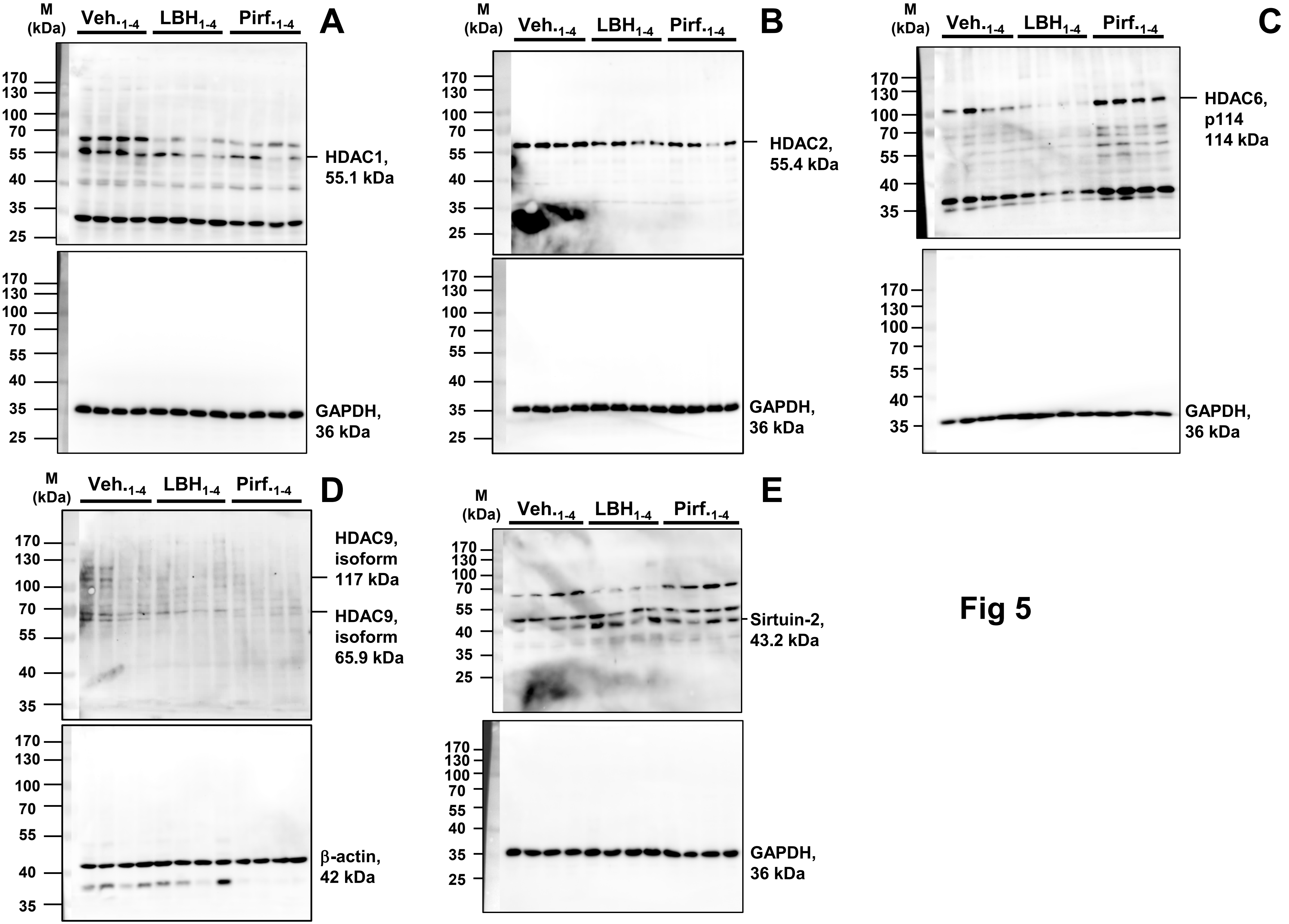

A
M (kDa)
Veh.1-4
LBH1-4
Pirf.1-4
170
130
100
70
55
40
35
25
HDAC1, 55.1 kDa
GAPDH, 36 kDa
B
HDAC2, 55.4 kDa
GAPDH, 36 kDa
C
HDAC6, p114 114 kDa
GAPDH, 36 kDa
D
HDAC9, isoform 117 kDa
HDAC9, isoform 65.9 kDa
β-actin, 42 kDa
E
Sirtuin-2, 43.2 kDa
GAPDH, 36 kDa

Fig 5

Fig 7

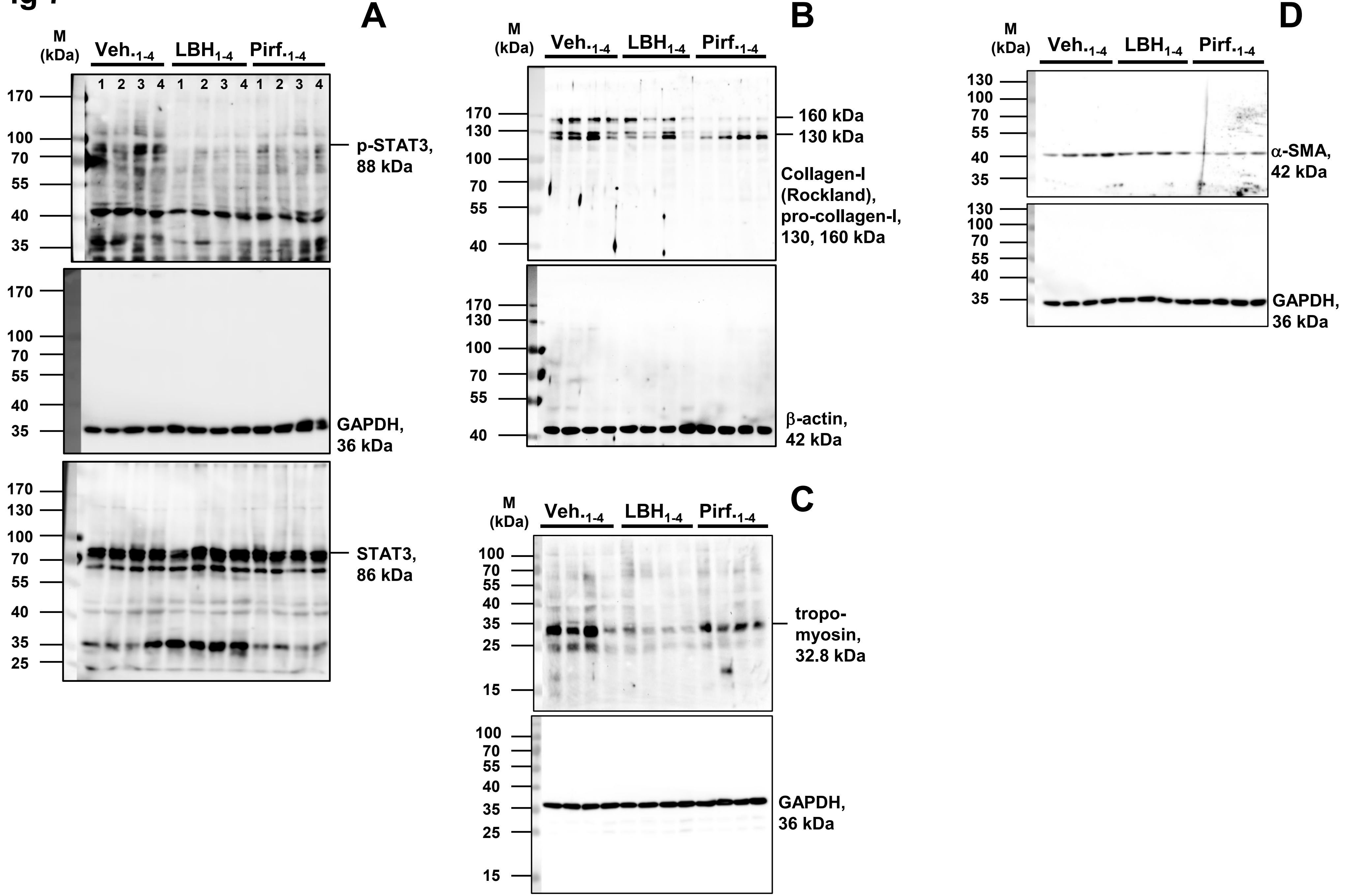
A
M (kDa)
Veh.1-4
LBH1-4
Pirf.1-4
1 2 3 4 1 2 3 4 1 2 3 4
170
100
70
55
40
35
p-STAT3, 88 kDa
GAPDH, 36 kDa
STAT3, 86 kDa
130
25
B
160 kDa
130 kDa
Collagen-I (Rockland), pro-collagen-I, 130, 160 kDa
β-actin, 42 kDa
C
tropo-myosin, 32.8 kDa
15
D
α-SMA, 42 kDa

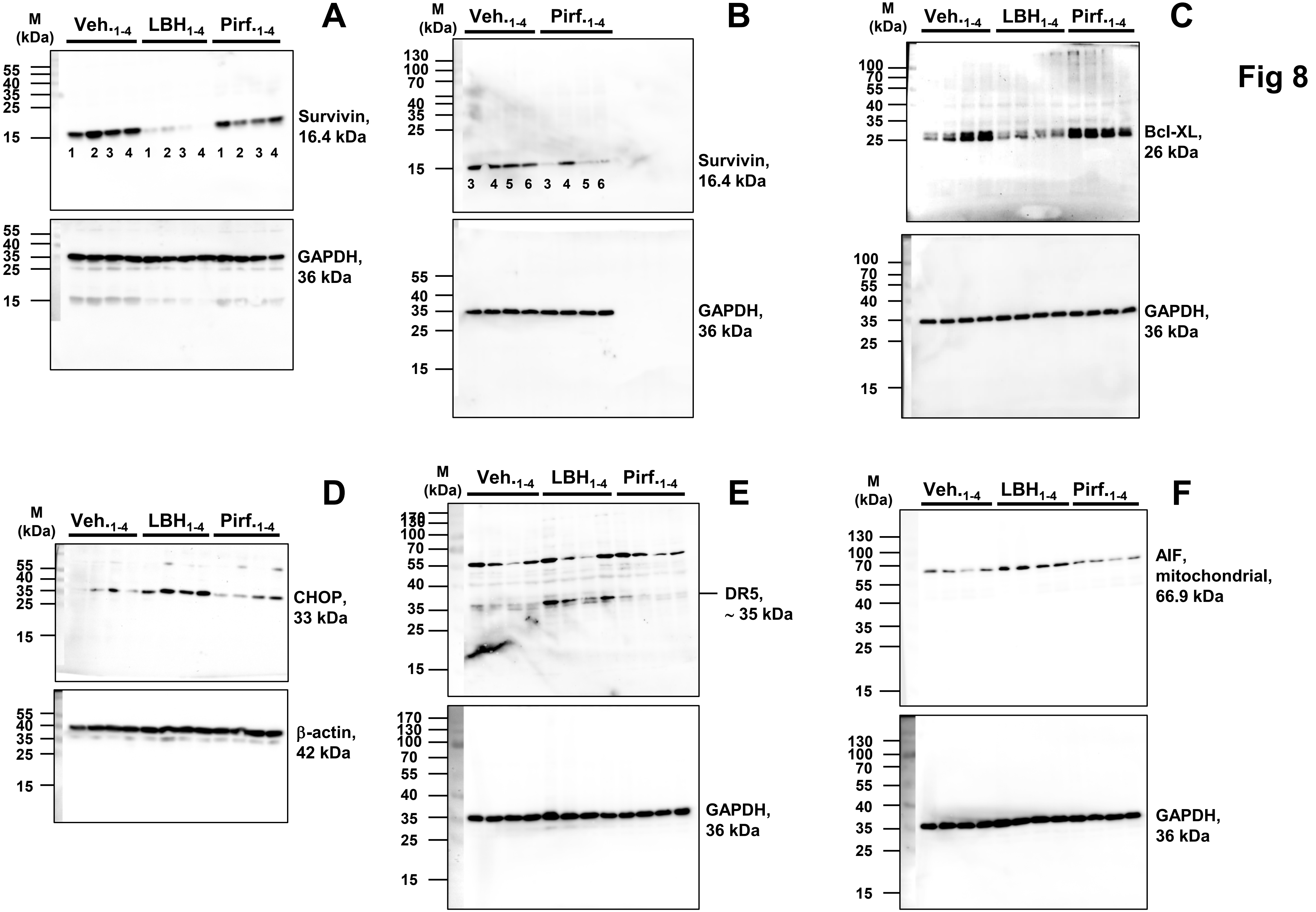

Fig 8
A
M (kDa)
Veh.1-4
LBH1-4
Pirf.1-4
Survivin, 16.4 kDa
GAPDH, 36 kDa
B
Veh.1-4
Pirf.1-4
Survivin, 16.4 kDa
GAPDH, 36 kDa
C
Veh.1-4
LBH1-4
Pirf.1-4
Bcl-XL, 26 kDa
GAPDH, 36 kDa
D
Veh.1-4
LBH1-4
Pirf.1-4
CHOP, 33 kDa
β-actin, 42 kDa
E
Veh.1-4
LBH1-4
Pirf.1-4
DR5, ~ 35 kDa
GAPDH, 36 kDa
F
Veh.1-4
LBH1-4
Pirf.1-4
AIF, mitochondrial, 66.9 kDa
GAPDH, 36 kDa

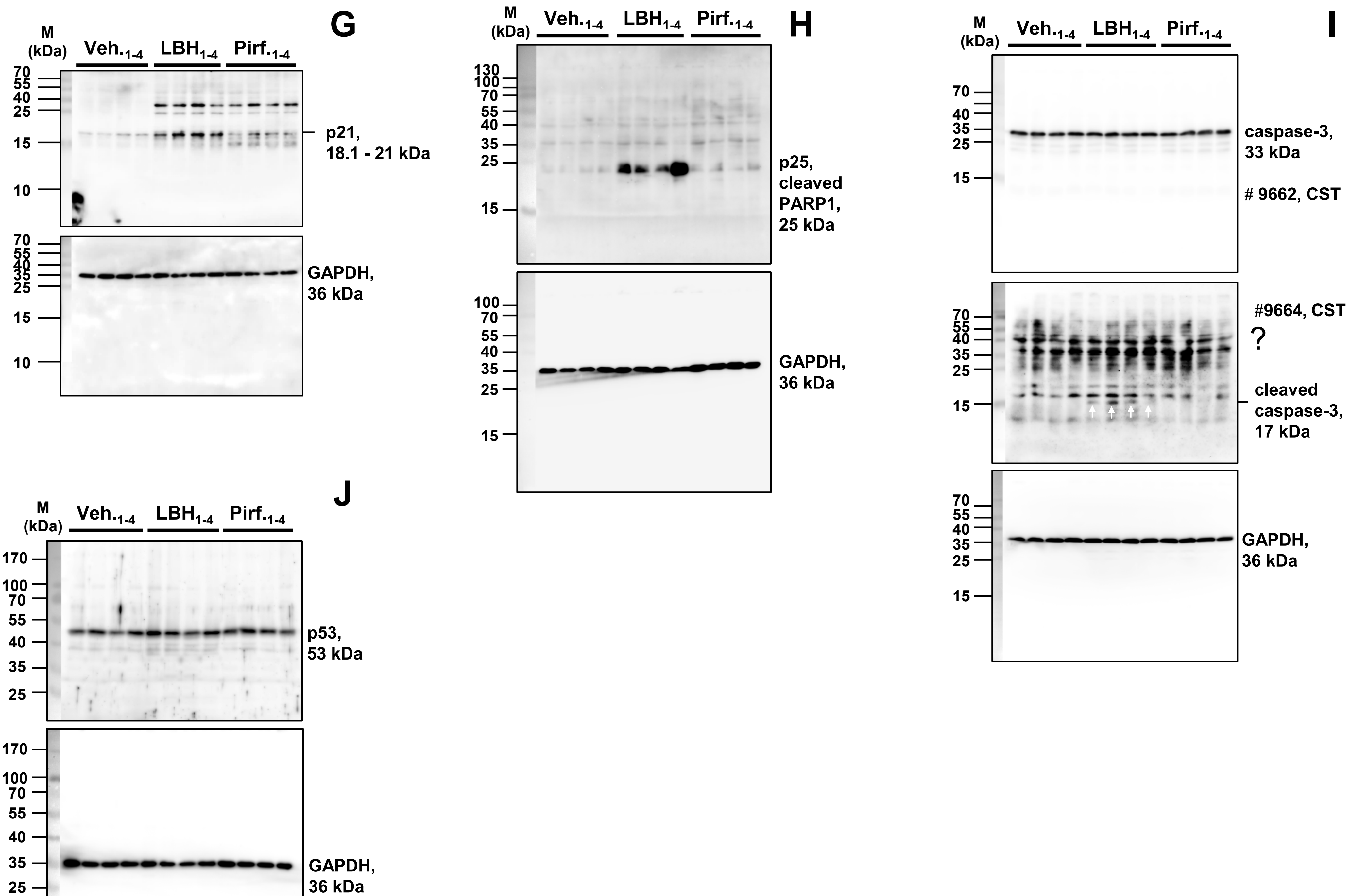

G
M (kDa)
Veh.1-4
LBH1-4
Pirf.1-4
70
55
40
25
15
10
p21,
18.1 - 21 kDa
70
55
40
35
25
15
10
GAPDH,
36 kDa
H
130
100
70
55
40
35
25
15
p25,
cleaved
PARP1,
25 kDa
100
70
55
40
35
25
15
GAPDH,
36 kDa
I
70
40
35
25
15
caspase-3,
33 kDa
9662, CST
#9664, CST
?
70
55
40
35
25
15
cleaved
caspase-3,
17 kDa
70
55
40
35
25
15
GAPDH,
36 kDa
J
170
100
70
55
40
35
25
p53,
53 kDa
170
100
70
55
40
35
25
GAPDH,
36 kDa

Fig 8

Fig 8

K

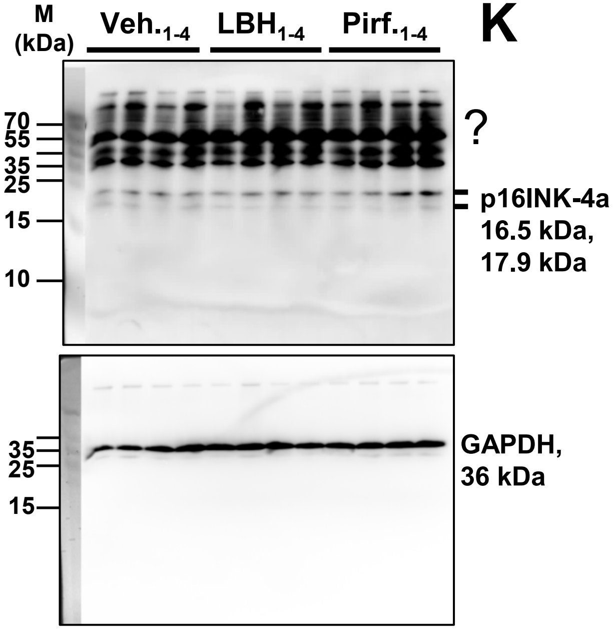

ab108349, Abcam, 2018

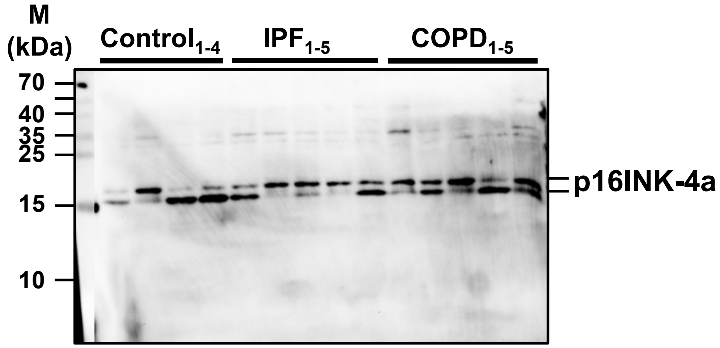

ab108349, Abcam, 2015

All blots with Page Ruler Prestained Protein Ladder, #26618, Thermo Fisher Scientific
